# Accuracy of ChatGPT, Gemini, Claude and DeepSeek in Carbohydrate Counting

**DOI:** 10.1111/dom.70747

**Published:** 2026-04-13

**Authors:** Luca Zagaroli, Nicholas Caione, Sabina Zara, Federica Guerra, Antonella Zugaro, Marco Giorgio Baroni, Maria Laura Iezzi, Maurizio Delvecchio

**Affiliations:** ^1^ Unit of Pediatrics, San Salvatore Hospital L'Aquila Italy; ^2^ Department of Biotechnological and Applied Clinical Sciences University of L'Aquila L'Aquila Italy; ^3^ Dietetics Service, San Salvatore Hospital L'Aquila Italy; ^4^ Diabetes Unit, San Salvatore Hospital L'Aquila Italy; ^5^ Department of Life, Health and Environmental Sciences University of L'Aquila L'Aquila Italy; ^6^ IRCCS Neuromed Pozzilli (IS) Italy

**Keywords:** artificial intelligence, carbohydrate counting, ChatGPT, Claude, DeepSeek, education, Gemini, nutrition, type 1 diabetes

## Abstract

**Aims:**

To evaluate the accuracy of four general‐purpose artificial intelligence (AI) models—ChatGPT (OpenAI), Gemini (Google), Claude (Anthropic) and DeepSeek (DeepSeek AI)—in calculating the carbohydrate content of meals compared with clinicians‐calculated reference values.

**Materials and Methods:**

The primary endpoint was equivalence between clinicians and AI‐generated calculations within an error margin of ±5%. One‐hundred twenty‐four meals were analysed, equally distributed among breakfast, lunch, dinner and snacks. Carbohydrate contents were jointly determined by two paediatric diabetologists and one clinical nutritionist using the USDA FoodData Central and CREA Italian Food Composition Tables. Each AI model received identical, standardized prompts in English describing the meals. Statistical analyses included the Two One‐Sided Tests procedure, the Bland–Altman plots, the Wilcoxon signed‐rank and the Spearman correlations.

**Results:**

The clinicians' median carbohydrate content was 30.32 g. Model medians were 30.75 g (ChatGPT), 30.40 g (Gemini), 29.75 g (DeepSeek) and 29.25 g (Claude). ChatGPT showed the smallest bias, the narrowest limits of agreement, and the highest correlation with clinicians' calculation. Only ChatGPT met the predefined ±5% equivalence criterion, whereas Gemini and DeepSeek achieved equivalence within a ±10% margin. Claude displayed the largest negative bias and the widest dispersion.

**Conclusions:**

ChatGPT most accurately approximated clinicians' carbohydrate calculation among the tested AI models and fulfilled strict clinical equivalence criteria. Although the other models tended to underestimate carbohydrate content, their mean deviations remained within clinically acceptable limits. These findings suggest that AI tools, particularly ChatGPT, may serve as useful adjuncts for carbohydrate counting for people with type 1 diabetes, supporting self‐management.

## 1 Introduction

Type 1 diabetes mellitus (T1DM) is characterized by autoimmune destruction of pancreatic β‐cells and an absolute insulin deficiency. Optimal management of people with T1DM requires a multidisciplinary approach that integrates insulin therapy, continuous glucose monitoring and medical nutritional therapy [1].

Carbohydrate counting plays a pivotal role in diabetes management as it allows tailoring prandial insulin doses to achieve glycemic targets [2]. Ideally, education on this aspect of management should be initiated as soon as possible, at the time of diagnosis in patients starting intensive insulin therapy, to promote accurate self‐management skills and support glycemic stabilization. Early education on carbohydrate counting has been associated with positive trends in glycemic control and lifestyle benefits, including improved glycated haemoglobin (HbA1c) levels, diabetes‐specific quality of life and coping ability in daily life [3]. Precision and reliability in carbohydrate calculation are key determinants of optimal postprandial glucose control and play a pivotal role in minimizing glycemic excursions [3]. Inaccurate carbohydrate calculation may lead to insulin mismatches, as underestimation of carbohydrates leads to hyperglycemia, while overestimation increases the risk of hypoglycemia [4, 5, 6], impairing the efficacy even of closed‐loop systems [4]. Despite these efforts and benefits, accurate carbohydrate counting remains a persistent challenge in routine diabetes management and providing simple and user‐friendly tools can improve calculation precision by reducing both under‐ and over‐estimation [3].

Previous studies have highlighted variability in artificial intelligence (AI) model performance when applied to healthcare settings [7]. Advancements in technology, including the growing integration of AI, particularly in the form of large language models (LLMs), combined with the widespread use of digital devices by youths with diabetes, their caregivers, and healthcare professionals, have created new opportunities in the field of nutritional support to enhance diabetes management. However, the clinical validity and accuracy of these tools in diabetes management have not yet been fully explored.

General‐purpose AI models such as ChatGPT (OpenAI) [8], Gemini (Google) [9], Claude (Anthropic) [10] and DeepSeek [11] can process natural language descriptions of meals and potentially assist in carbohydrate calculation.

The aim of this study was to evaluate the accuracy of AI models in calculating meal carbohydrate content compared with clinician‐derived reference values, and to explore their potential role as supportive tools in carbohydrate counting. This research seeks to inform the potential application of AI‐assisted carbohydrate counting in diabetes care to provide a further useful tool for management.

## 2 Materials and Methods

### 2.1 Endpoint

The primary endpoint was to assess equivalence between AI‐generated carbohydrate estimates and the clinicians‐derived reference values. To achieve this aim, we evaluated the accuracy of AI models in calculating the carbohydrate content of meals using precise descriptions.

### 2.2 Study Design

This study was conducted with four general‐purpose AI models: ChatGPT‐5.0 (OpenAI), Gemini 2.5 Flash (Google), Claude 4 Sonnet (Anthropic) and DeepSeek R1 (DeepSeek AI). The AI‐generated calculations were compared against reference values calculated by three clinicians using two validated nutrition databases, the USDA FoodData Central [12] and the CREA Italian Food Composition Tables [13]. The reference carbohydrate content of each meal was manually calculated jointly by consensus by two paediatric diabetologists (M.D. and L.Z.) and one nutritionist (S.Z.), with final values defined through consensus discussion.

The four models were selected based on their widespread public availability, robust language understanding capabilities, and relevance in clinical and consumer settings. Unlike research‐focused or code‐oriented models (e.g., Mistral), these general‐purpose LLMs were representative of the tools that could feasibly be integrated into routine patient support, including those with limited technological or linguistic skills. Their inclusion was based not only on technical performance, but also on accessibility, interface maturity, and user‐friendliness, especially for non‐specialist users. A detailed summary of the prompting environment and model identifiers as displayed in the respective interfaces is provided in Table S1.

The required sample size for the primary endpoint was determined a priori through power analysis. A very narrow equivalence margin of ±5% was adopted, reflecting the objective of identifying the model that best approximates clinicians' assessment. Under this assumption, a minimum of 124 paired observations was calculated to ensure 80% statistical power at an alpha level of 0.05. This sample size was considered sufficient to robustly evaluate equivalence between AI and clinicians' calculation [14]. Meals were manually created specifically for this study and equally distributed among four categories: breakfast, lunch, dinner and snacks (31 meals per category). Each meal was transcribed in a precise descriptive format, including explicit food items and quantities (e.g., ‘80 g of whole wheat pasta with 10 g of olive oil’) (Table S2). The dataset intentionally included meals with very low or negligible carbohydrate content in order to evaluate model performance across a broad spectrum of food compositions, including meals in which correct identification of minimal carbohydrate content was required. The meal composition was developed based on our experience about patients' reported dietary habits.

### 2.3 LLM Configuration and Prompting Environment

All LLM models were accessed through their respective web‐based interfaces using the Google Chrome browser on desktop computers, without logging into a personal user account. No application programming interfaces (APIs) were used.

The evaluated models were ChatGPT‐5.0 (OpenAI), Gemini 2.5 Flash (Google), Claude 4 Sonnet (Anthropic) and DeepSeek R1 (DeepSeek AI), as identified by the model names displayed in the respective web interfaces at the time of prompting. All prompts were submitted in English from computers physically located in Italy. No virtual private network (VPN) or location‐masking tools were used. The initial prompting phase for all four models was conducted between October and November 2025.

To minimize contextual carryover between prompts, each meal description was evaluated within a newly created conversation (‘new chat’). After each prompt submission, the conversation was closed and a new chat was initiated before entering the next meal description. No iterative or follow‐up prompts were used, and each meal was assessed independently using an identical, pre‐specified prompt.

When multiple outputs were generated for the same meal description using the same LLM, only one predefined output per meal was retained for statistical analysis. Specifically, the output generated during the final prompting session using the most recent model version was selected, in order to ensure consistency across meals and to avoid pseudo‐replication.

Because the web‐based interfaces were accessed without user authentication, no persistent user memory or account‐level personalization features were available during prompting. However, given the proprietary nature of commercial LLM platforms, the possibility of implicit system‐level personalization cannot be entirely excluded and is acknowledged as an inherent limitation of real‐world LLM evaluations.

Prompts were carefully designed to require calculation based solely on food composition, without inference. Each AI received the same instruction in English, formatted as follows: ‘Please estimate the total amount of carbohydrates (in grams) in the described meal. This estimation is intended for a patient with type 1 diabetes mellitus. Use standard nutritional databases, consider raw weights unless otherwise specified, and do not make assumptions about ingredients not explicitly mentioned’.

### 2.4 Statistical Analysis

Descriptive statistics were computed for each model, including mean and standard deviation (SD), median and interquartile range (IQR), SD of differences, mean absolute error and signed bias. The distribution of errors was tested using the Shapiro–Wilk test for normality, showing a non‐normal distribution for the study variables. The predefined equivalence margin was ±5% of the clinicians'‐derived mean. To assess systematic differences, Wilcoxon signed‐rank tests were applied. Bland–Altman plots were generated to assess per‐meal agreement between each AI model and the clinicians' reference. These plots visualize the mean bias and the 95% limits of agreement (±1.96 SD), offering a graphical representation of dispersion and systematic deviation. Equivalence was formally tested using the Two One‐Sided Tests (TOST) procedure. Spearman correlation coefficients were computed to evaluate linear association between clinicians and AI calculation. To assess whether AI performance deteriorated with higher‐carbohydrate meals, we conducted an exploratory analysis correlating reference values with absolute and relative calculation errors for each model. Spearman correlation coefficients (*ρ*) and two‐sided *p*‐values were calculated. This analysis evaluated the potential for range‐restriction bias, whereby smaller meals might artificially inflate model performance. As clinically interpretable secondary endpoints, we calculated for each model the proportion of meals with an absolute error within ±5, ±10 and ±15 g relative to the clinicians' reference value, as well as the proportion of meals with an absolute error > 10 g. As an additional clinical translation analysis, carbohydrate‐counting errors were converted into theoretical insulin‐dose differences using insulin‐to‐carbohydrate ratios of 1:8, 1:10 and 1:15. We also calculated the proportion of meals with carbohydrate overestimation > 10 g and the proportion of meals associated with a theoretical excess insulin dose > 0.5 and > 1.0 U. These analyses were performed both in the overall dataset and in the subgroup of meals containing > 50 g of carbohydrates. Meals were also stratified according to the clinicians' reference carbohydrate content into three predefined groups: < 20, 20–50 and > 50 g.

Statistical analysis was performed with IBM SPSS Statistics version 30.x. Global between‐model differences in the proportion of meals within predefined absolute‐error thresholds were assessed using Cochran's Q test. When appropriate, pairwise post hoc comparisons were performed using McNemar exact tests with Bonferroni correction. Between‐model differences in absolute error were assessed using the Friedman test. TOST procedure was performed using Python 3.x with the statsmodels package. Differences were considered statistically significant if *p*‐value < 0.05.

### 2.5 Ethics Statement

This study used standardized hypothetical meal descriptions and did not involve human participants, identifiable patient data, or biological samples; therefore, ethics committee approval and informed consent were not required.

## 3 Results

### 3.1 Descriptive Analysis

A total of 124 meals were analysed. The clinicians' median carbohydrate calculation was 30.32 (20.38–42.74) g. Model medians were 30.75 (20.15–40.80) g (ChatGPT), 30.40 (20.06–39.55) g (Gemini), 29.75 (20.00–40.87) g (DeepSeek) and 29.25 (20.60–38.50) g (Claude). The smallest bias was observed for ChatGPT, while Gemini and DeepSeek showed modest negative biases, and Claude showed the largest negative bias. Consistently, the SD of differences was narrowest for ChatGPT and wider for Gemini, DeepSeek and Claude, indicating progressively lower per‐meal precision across these models (Table 1).

**TABLE 1:** Summary metrics per model: Mean ± standard deviation (SD), median (interquartile range—IQR), SD of differences (AI−Clinicians), absolute error, bias, Wilcoxon test results, Rho di Spearman, 95% limits of agreement (LoA).

Model	Mean ± SD (g)	Median (IQR) (g)	SD diff (g)	Abs error (g)	Bias (g)	Wilcoxon p	Spearman rho	95% LoA lower/95% LoA upper
Clinicians	33.71 ± 19.55	30.32 (20.38–42.74)						
ChatGPT	33.41 ± 18.97	30.75 (20.15–40.80)	3.82	2.71	−0.302	0.784	0.970	−7.799/7.196
Gemini	31.90 ± 16.28	30.40 (20.06–39.55)	10.23	4.79	−1.811	0.894	0.896	−21.863/18.241
DeepSeek	32.59 ± 17.69	29.75 (20.00–40.87)	10.71	5.46	−1.123	0.422	0.887	−22.122/19.876
Claude	31.16 ± 16.87	29.25 (20.60–38.50)	11.57	6.03	−2.554	0.536	0.856	−25.238/20.129

### 3.2 Pairwise Comparisons With the Clinicians

We conducted Wilcoxon signed‐rank tests on meal‐level differences (AI−Clinicians). None of the AI models showed evidence of a systematic difference from the clinicians‐derived reference values (Table 1).

### 3.3 Agreement Analysis

**FIGURE 1:**
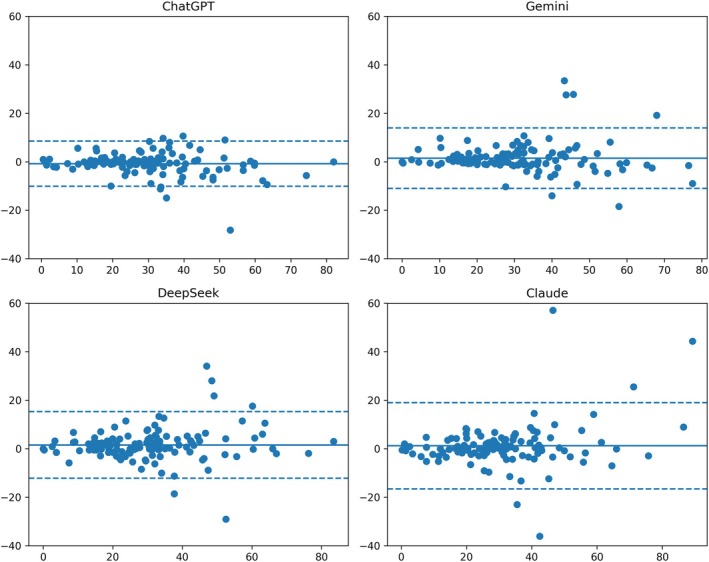
Bland–Altman plots showing agreement between carbohydrate estimates generated by AI models (ChatGPT, Gemini, DeepSeek and Claude) and reference values calculated by clinicians. The *y*‐axis represents the difference between the model estimate and the reference value (g), while the *x*‐axis represents the mean of the model estimate and the reference value (g). Solid lines indicate mean bias, and dashed lines indicate the 95% limits of agreement.

**FIGURE 2:**
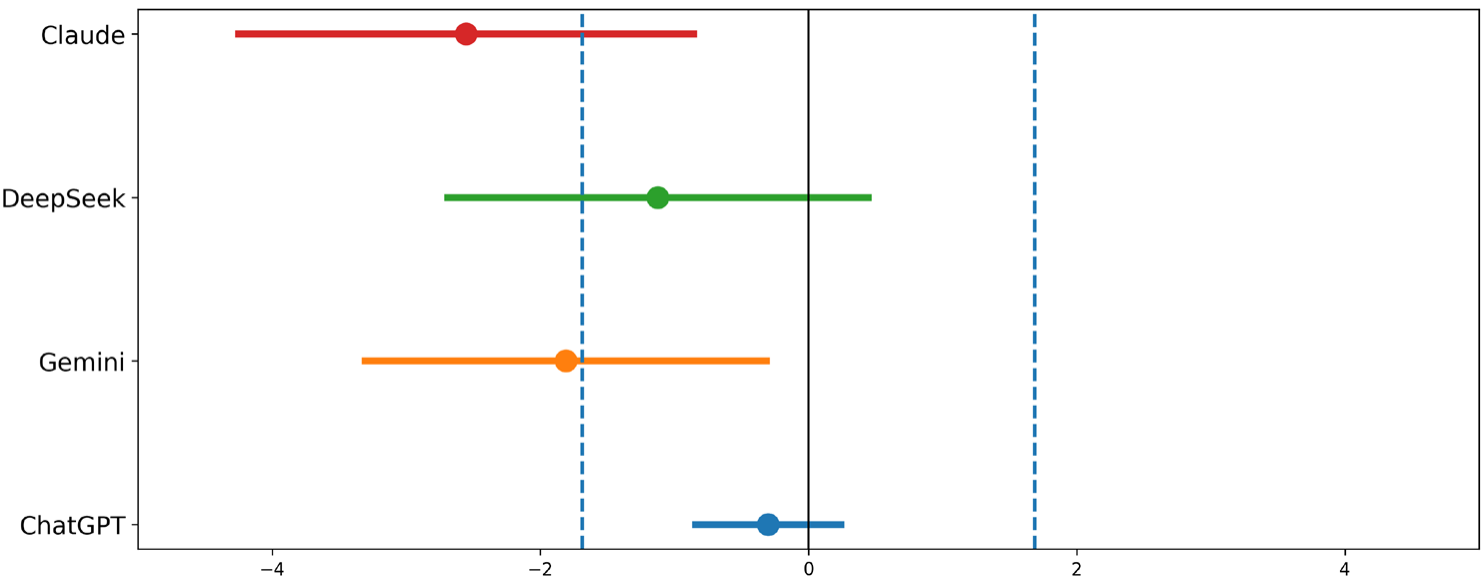
Equivalence analysis of AI‐generated carbohydrate estimates compared with reference values. The *x*‐axis represents the mean difference (model − reference value, g) and the *y*‐axis the evaluated AI models; points indicate mean differences and horizontal lines 90% confidence intervals. Dashed vertical lines denote the equivalence margins (±5% of the mean reference value), used to explore potential statistical equivalence.

Bland–Altman plots showed the smallest bias and the tightest limits of agreement for ChatGPT, indicating the best per‐meal precision and minimal systematic error (Figure 1; bias, SD of differences, and 95% limits in Table 1). Gemini, DeepSeek and Claude displayed wider limits of agreement, consistent with greater dispersion of errors and reduced precision at the single‐meal level. Figure 2 summarizes equivalence testing by plotting mean differences with 90% confidence intervals (CIs) against the ±5% bounds. Only ChatGPT lies entirely within the equivalence region, whereas Gemini, DeepSeek and Claude cross at least one bound.

### 3.4 Equivalence Testing

Equivalence was evaluated using a pre‐specified ±5% margin relative to the clinicians' calculation. Only ChatGPT met the ±5% equivalence criterion, with the 90% CI of the mean difference entirely contained within the bounds (Table 1; Figure 2). Gemini, DeepSeek and Claude did not meet equivalence at ±5% because their CIs extended beyond at least one bound. To assess whether widening the error margin would allow the evaluated models to be statistically equivalent to the reference values, a further sensitivity analysis using a ±10% equivalence margin showed that ChatGPT, Gemini and DeepSeek met equivalence (both one‐sided TOSTs significant), whereas Claude did not (failure of the lower one‐sided test) (Figure S1). Given its clinical relevance, we consider this result noteworthy and report it here for completeness.

### 3.5 Clinically Interpretable Error Thresholds

As a secondary analysis based on absolute error in grams, ChatGPT showed the highest proportion of meals within clinically relevant thresholds: 82.3% of meals were within ±5 g, 98.4% within ±10 g, and 100.0% within ±15 g of the clinicians' reference, with only 1.6% of meals showing an absolute error > 10 g. Respectively, the corresponding values were 78.2%, 91.1%, 93.5% and 8.9% for Gemini; 74.2%, 89.5%, 91.9% and 10.5% for DeepSeek; and 73.4%, 88.7%, 91.1% and 11.3% for Claude. Across the overall dataset, the proportion of meals within ±10 g differed significantly between models (*p* = 0.001). In pairwise post hoc analyses, ChatGPT showed a significantly higher proportion of meals within ±10 g than DeepSeek and Claude after Bonferroni correction (Table S3).

### 3.6 Clinical Translation of Carbohydrate‐Counting Error

A pragmatic translation of carbohydrate‐counting error into theoretical insulin‐dose differences is reported in Table S4. Across insulin‐to‐carbohydrate ratios of 1:8, 1:10 and 1:15, ChatGPT showed the lowest mean theoretical dose difference both overall and in meals containing > 50 g of carbohydrates, whereas larger discrepancies were observed for Gemini, DeepSeek, and especially Claude. In the overall dataset, carbohydrate overestimation > 10 g was uncommon for all models but occurred more frequently in DeepSeek and Claude than in ChatGPT. In the > 50 g subgroup, between‐model differences were mainly driven by underestimation for Gemini and DeepSeek, whereas Claude showed both greater overall variability and the highest proportion of meals associated with larger theoretical excess insulin doses.

### 3.7 Correlation With the Reference Values

Spearman correlations between model calculations and the clinicians were high overall (Table 1; Figure S2). *p*‐value was < 0.001 for all the correlation tests but ChatGPT showed the strongest linear agreement, followed by Gemini, DeepSeek and Claude. Thus, while all models tracked the general variability of meal carbohydrate content, ChatGPT most closely mirrored the clinicians' pattern.

### 3.8 Exploratory Correlation Analysis

We evaluated whether absolute calculation errors increased with higher‐carbohydrate meals. Absolute error showed weak positive correlations with clinicians' carbohydrate content for all AI models (*ρ* = 0.28–0.36, all *p* < 0.01), consistent with a small but statistically significant increase, not clinically meaningful, in error for larger meals. ChatGPT exhibited the lowest correlation (*ρ* = 0.277), consistent with the most stable performance across carbohydrate range (Figure S3).

### 3.9 Stratified Analysis by Carbohydrate Load

When meals were stratified according to the clinicians' calculation, all models performed well in the < 20 g stratum, where the proportion of meals within ±10 g ranged from 96.7% to 100.0%, without any significant between‐model difference (*p* = 0.392). In the 20–50 g stratum, performance remained generally high, with ChatGPT showing the best profile (82.9% of meals within ±5 g and 98.6% within ±10 g), again without significant between‐model differences (*p* = 0.305). In contrast, differences emerged in the > 50 g subgroup. ChatGPT retained the best performance (62.5% of meals within ±5 g, 95.8% within ±10 g, mean absolute error 4.00 g). By contrast, Gemini and DeepSeek each showed 29.2% of meals with an absolute error > 10 g and Claude 45.8% and a mean absolute error of 18.50 g. In this stratum, the proportion of meals within ±10 g differed significantly across models (*p* < 0.001). In pairwise post hoc analyses, ChatGPT performed significantly better than Claude after Bonferroni correction and showed better performance than Gemini and DeepSeek, but not statistically different (unadjusted *p* = 0.07 for both comparisons; Bonferroni‐adjusted *p* = 0.422 for both) (Table S3).

### 3.10 Error‐Categorization Checklist for ChatGPT

We conducted the error checklist only for ChatGPT, as it was the most statistically reliable model and therefore the most clinically pertinent target for qualitative error analysis. Across the 124 meals, we identified 30 annotated errors. The main categories were: reference discrepancy (clinicians/database alignment) (33.3% of all annotations; 8.1% of meals), generic/average values without brand‐specific matching (20.0%; 4.8% of meals), quantitative over/underestimation for specific items (16.7%; 4.0% of meals), other/unspecified (16.7%; 4.0% of meals), brand information not used (6.7%; 1.6% of meals), instructions not strictly followed (3.3%; 0.8% of meals), and raw versus cooked mismatch (3.3%; 0.8% of meals). Notably, a subset of notes flagged potential reference‐side inconsistencies (e.g., clinicians/database reconciliation), reminding that small uncertainties in the gold standard can influence per‐meal error attribution (Table S5).

## 4 Discussion

This study suggests that AI, particularly ChatGPT, may serve as a useful supportive tool for carbohydrate counting in T1DM under standardized conditions. We evaluated the performance of four general‐purpose AI models—ChatGPT (OpenAI), Gemini (Google), Claude (Anthropic) and DeepSeek (DeepSeek)—in calculating the carbohydrate content of meals reported in a precise descriptive format as compared to clinicians‐calculated values. Within this condition, ChatGPT (OpenAI) showed the best agreement with the clinicians' reference as it demonstrated the most accurate (±5% margin) and consistent performance across all evaluation metrics, the lowest mean absolute error, the smallest bias, the lowest standard deviation of errors, and the highest correlation coefficient. Furthermore, it produced the narrowest limits of agreement in Bland–Altman analysis. A further sensitivity analysis showed that Gemini and DeepSeek met equivalence with the clinicians' calculation using a ±10% equivalence margin, whereas Claude did not (Figure S1). All these findings suggest that ChatGPT counting was the most accurate model on average, the most consistent on a per‐meal basis, and thus the most reliable AI model for potential use as an adjunctive aid in carbohydrate counting.

These findings are in line with recent literature supporting the role of AI as a complementary tool in the management of T1DM. AI may facilitate self‐management, education and active patient involvement, improving therapy adherence and quality of life [15, 16, 17, 18], and may potentially reduce disparities related to health literacy and caregiver experience [19, 20]. As regards nutrition, studies evaluating LLM models for dietary assessment reported good performance in food identification and meal ranking, but variable accuracy in portion size and nutrient estimation, particularly for complex meals [21, 22]. Investigations have also suggested that image‐based AI models may offer complementary strengths—accurately recognizing food items yet tending to underestimate portion sizes in larger meals [22]. Comparisons with dietitians showed moderate agreement and systematic biases, underscoring the need for caution when applying AI‐generated calculates to insulin dosing decisions [23].

Accuracy in carbohydrate counting is crucial, as it is strongly associated with better postprandial glycemic control. Our paper shows that AI models may properly support patients and caregivers in this task, but only ChatGPT met the predefined margin of ±5% as compared to clinicians' calculation. Even if results are heterogeneous and benefits may vary by individual and family context, systematic reviews and meta‐analyses show that carbohydrate counting reduces HbA1c. The average reduction ranges from 0.35% to 0.55% compared to standard dietary advice or other meal planning methods, with the effect being more pronounced in adults [24, 25, 26, 27, 28]. Underestimation leads to hyperglycemia, while overestimation increases hypoglycemia risk. To overcome carbohydrate counting inaccuracy and to reduce the distress of diabetes burden for patients and caregivers, recently simplified meal announcement (using preset carbohydrate amounts) has been proposed as an alternative approach overall for those adolescents using advanced insulin pump systems who struggle with precise counting. However, randomized controlled trials found that precise carbohydrate counting results in a higher percentage of time spent in the target glucose range (TIR) compared to simplified meal announcements, even when both groups met international glycemic targets [29, 30]. As well, telemedicine and app‐based interventions for carbohydrate counting showed significant improvements in HbA1c and TIR, confirming the benefit of precise carbohydrate counting across different delivery methods [28, 31].

Notably, errors above approximately 10 g have been associated with worse glycemic outcomes in some settings [32], although their clinical impact may vary according to the insulin‐to‐carbohydrate ratio, the clinical context and pre‐meal glycemic conditions. From a clinically pragmatic perspective, the proportion of meals within ±10 g of the clinicians' reference remained highest for ChatGPT (98.4%), and lower for Gemini (91.1%), DeepSeek (89.5%) and Claude (88.7%). Thus, although only ChatGPT met the prespecified ±5% equivalence criterion, Gemini and DeepSeek may still offer some practical support for carbohydrate counting when performance is interpreted using clinically meaningful absolute‐error thresholds rather than formal equivalence alone.

A pragmatic clinical translation further supported this interpretation. When carbohydrate‐counting error was converted into theoretical insulin‐dose differences, ChatGPT consistently showed the lowest mean dose discrepancy, whereas Gemini, DeepSeek, and particularly Claude showed larger potential deviations. These findings do not allow direct estimation of glycemic outcomes or hypoglycemia frequency, but they provide an additional clinically interpretable indication of the potential impact of model variability, especially in meals with higher carbohydrate content. In addition, lower variability (SD and IQR) in AI models may reduce the likelihood of large estimation errors that could result in excessive insulin dosing. Importantly, the observed negative bias indicates a tendency towards underestimation rather than overestimation, suggesting a limited theoretical risk of hypoglycemia. Nonetheless, these findings should be interpreted cautiously, as real‐world risk depends on additional clinical factors.

Carbohydrate‐counting education should also be individualized in paediatric practice. In very young children, during prolonged partial remission, or in families under marked emotional stress, introducing highly detailed carbohydrate counting too early may increase burden without clear short‐term benefit. In such cases, a stepwise approach may be preferable, with gradual progression to more precise carbohydrate counting when clinically and psychologically appropriate.

In clinical practice, larger and more complex meals (e.g., pasta, rice, mixed dishes, potatoes) are associated with greater errors, most in patients with lower numeracy skills or less experience [33, 34]. Errors are more pronounced in children, adolescents, and with larger meal sizes [33, 35]. Similarly, our data showed that absolute calculation error increased with higher carbohydrate content, although the correlations were weak in magnitude (*ρ* < 0.36), indicating only a minimal increase in error for larger meals. Meals were created specifically for this study, and the relatively low median carbohydrate content of our dataset might appear as a sampling limitation. Some low‐carbohydrate or nearly carbohydrate‐free meals were intentionally included to evaluate model behaviour across a broad range of food compositions, including situations in which correct recognition of minimal carbohydrate content was required. However, the stratified analysis confirmed that performance differences became more evident as carbohydrate load increased, particularly for meals > 50 g, in which ChatGPT remained the most robust model whereas Gemini, DeepSeek, and especially Claude showed a substantially higher proportion of errors > 10 g.

The qualitative review of ChatGPT's errors revealed omissions of ingredients, confusion between raw and cooked weights, and misinterpretation of portion sizes. Many discrepancies appear preventable through more specific user inputs and basic guardrails. Common issues included reliance on generic averages rather than product‐specific values, missing brand or label information and raw‐versus‐cooked ambiguities. Fruit items—particularly bananas—were a recurrent source of variability due to differences in variety and ripeness affecting sugar content. Some discrepancies also reflected legitimate reference‐side choices, such as database selection or rounding, highlighting that small uncertainties in the reference standardized lookup rules may substantially reduce per‐meal error when AI tools are used for carbohydrate counting.

This study has several limitations that should be considered when interpreting the findings. First, it could be debated that our conclusions could only be applied to a specific carbohydrates content range. Such potential methodological concern is justified by range‐restriction bias, whereby in our dataset some meals (snacks) did not contain carbohydrates, but this point has been previously discussed. A necessary next step will be to replicate this study in the context of larger meals with higher carbohydrate loads, to extend the applicability of our conclusions to older or larger patient populations. Second, general‐purpose LLMs are inherently stochastic and are periodically updated. Even with fixed prompts, run‐to‐run and model‐version variability may introduce subtle differences in output. Third, we evaluated standardized, text‐only meal descriptions; real‐world inputs are often briefer or ambiguous (brands, cooking methods, sauces, mixed dishes), so generalizability beyond this controlled setting should be confirmed. Fourth, the meal spectrum, although structured, may not fully reflect the diversity of free‐living diets or culturally specific recipes.

A key strength of this study lies in the use of a standardized dataset benchmarked against clinicians' calculations and a comprehensive statistical framework capturing both accuracy and precision. The findings suggest that performance and clinical utility could be further improved through pragmatic measures, including light calibration and guardrails, retrieval from validated nutrition databases, consistent source conditioning, and domain adaptation to diabetes‐specific use cases (Table 2). However, real‐world implementation will require validation on patient‐generated inputs, which are often incomplete or ambiguous, and consideration of contextual factors such as literacy, cultural habits and food preparation variability. In addition, although English prompts were used to standardize model interaction, this choice may have reduced brand‐specific matching or cultural food mapping for some Italian foods. Within‐version variability across repeated runs was not directly assessed in the present study. Notably, AI models performed more accurately with standardized or branded foods, highlighting current limitations in estimating non‐standardized, home‐cooked meals.

**TABLE 2:** Practical issues for carbocounting with AI‐models.

Input specificity Brand/label: always include brand and product line (e.g., ‘Oro Saiwa wholemeal biscuits, 30 g’). Fruit details: specify variety and ripeness stage (e.g., for bananas specify Cavendish, ripe/yellow with few spots; for apples specify Golden/Granny Smith). Raw versus cooked: state it explicitly for pasta, rice, couscous, potatoes, vegetables, meat (‘80 g pasta raw’ vs. ‘200 g cooked rice’). Portion units: prefer grams or milliliter; avoid pieces/slices unless you give the weight per piece. Add‐ons and sauces: list them with amounts (oil, cheese, cream cheese, jam, dressings). Packaged items: add serving size and label carbs/100 g if known.
Retrieval and mapping Validated databases: require using CREA/USDA database and cite the chosen entry. Disagreement rule: pre‐specify a tie‐breaker (e.g., ‘use the entry with closer product specificity; otherwise default to CREA for Italian items, USDA otherwise’). No generic averages by default: allow ‘average values’ only as a last resort and flag them in output.
Guardrails in the prompt Instruct the model to: List matched database items and their carbs/100 g, Warn when brand/variety/ripeness is missing (‘Result may vary due to unspecified variety/ripeness’). Enforce net versus total carbs convention (pick one; be explicit and consistent).
Calibration and QA Bias correction: apply a simple offset if a recurrent bias seems to occur Spot‐checks: for high‐impact categories (pizza, couscous, instant barley, biscuits, juices), cross‐verify a random subset against the label/database. Template use: collect meals via a structured template (fields for brand, variety, ripeness, raw/cooked, weights, sauces).

This issue is important for pragmatic implementation. In routine care, less structured meal descriptions may reduce accuracy rather than improve it, particularly when relevant details on portion size, ingredients or preparation are missing. Therefore, AI‐assisted carbohydrate counting should currently be viewed as an adjunct to, rather than a replacement for, foundational diabetes education. Its performance in real‐world patient‐ or caregiver‐generated inputs requires dedicated prospective evaluation. This interpretation is consistent with a broader human‐in‐the‐loop perspective, in which AI tools are used to support, rather than replace, clinical reasoning and patient education. Similar collaborative‐intelligence frameworks have been advocated across different areas of clinical AI, emphasizing that technological gains are most reliable when integrated with human expertise, contextual oversight and structured implementation pathways [36, 37, 38]. In this framework, AI may improve access and consistency, but human supervision remains essential for contextual judgement and safe implementation. Moreover, these findings may not transfer identically to future model versions or to real‐world use through personalized accounts, mobile applications or mixed conversational prompts, all of which may modify output behaviour through contextual carryover or interface‐related differences.

Recent evidence has explored AI‐based carbohydrate estimation in culturally specific food settings, supporting the growing clinical relevance of this field while highlighting persistent variability across meal types and platforms [39].

In this context, the present study adds clinically relevant information to a still limited literature by providing a head‐to‐head evaluation of multiple general‐purpose LLMs against a clinicians‐derived reference standard in a paediatric diabetes‐oriented setting, and by combining formal equivalence testing with clinically interpretable absolute‐error thresholds and qualitative error categorization. Unlike prior reports based on single‐model assessments, image‐based estimation or different food contexts, the present study provides a structured head‐to‐head benchmark of multiple general‐purpose LLMs under standardized prompting conditions. Future studies should extend this approach to less structured real‐world inputs, infant and small‐meal scenarios, and multimodal assessment strategies integrating both text and image‐based inputs. These findings should also be interpreted in the context of the growing number of smartphone applications and digital tools that estimate nutritional composition from food images (e.g., GoCARB, SNAQ, RxFood). These systems analyse meal photographs to identify foods and estimate portion size and macronutrient composition. They may further support carbohydrate counting and are increasingly recognized as important components of technology‐supported diabetes care and self‐management [40, 41, 42, 43]. Compared with these tools, the present study focused specifically on text‐based, general‐purpose LLMs evaluated under standardized prompting conditions. The integration of text‐based and image‐based approaches may represent a relevant future direction for clinically applicable AI‐assisted carbohydrate counting. The same framework may also support periodic re‐evaluation of evolving commercial models, using fixed benchmark meals, standardized prompts, transparent reporting of model version and access conditions and prespecified agreement and error‐threshold metrics.

## 5 Conclusions

AI tools may serve as valuable tools in carbohydrate counting for patients when appropriate description is provided, with a precision comparable to clinicians' estimations. ChatGPT is the one that performs best in carbohydrate counting compared to the clinicians among general‐purpose AI models. The other models tend to slightly underestimate the clinicians' reference with wider dispersion of errors, but the errors appear to be within a clinically acceptable limit.

Future studies should include a broader range of high‐carbohydrate meals to fully characterize model behaviour across different dietary conditions, real‐life patient data, assess the impact of AI‐supported carbohydrate counting on glycemic outcomes, and explore the integration of AI into hybrid human‐AI workflows that prioritize safety, transparency and accountability.

## Author Contributions

**Luca Zagaroli:** conception and design of the work. **Nicholas Caione** and **Federica Guerra:** drafting the article. **Sabina Zara:** acquisition, analysis or interpretation of data for the work. **Antonella Zugaro:** reviewing the article critically for important intellectual content. **Marco Giorgio Baroni:** final approval of the version to be published. **Maria Laura Iezzi:** reviewing the article critically for important intellectual content. **Maurizio Delvecchio:** guarantor of the paper, final approval of the version to be published. All the authors read and approved the final manuscript.

## Funding

The authors have nothing to report.

## Conflicts of Interest

The authors declare no conflicts of interest.

## Supporting information


**Figure S1:** Equivalence analysis of AI‐generated carbohydrate estimates compared with reference values. The *x*‐axis represents the mean difference (model − reference value, g) and the *y*‐axis the evaluated AI models; points indicate mean differences and horizontal lines 90% confidence intervals. Dashed vertical lines denote the equivalence margins (±10% of the mean reference value), used to explore potential statistical equivalence.

**Figure S2:** Scatter plots comparing AI‐generated carbohydrate calculations with reference values calculated by clinicians. Each panel represents one AI model. The *x*‐axis shows the reference value (g) and the *y*‐axis the corresponding AI estimate (g), using the same scale across all panels. The diagonal line indicates the line of identity (*y* = *x*), representing perfect agreement.

**Figure S3:** Scatter plots of AI‐generated carbohydrate calculations versus clinicians' calculation values with relative error bands. The *x*‐axis represents the reference value (g) and the *y*‐axis the corresponding AI estimate (g), using the same scale across all panels. The solid diagonal line indicates the line of identity (*y* = *x*), while the additional diagonal lines represent ±5%, ±10% and ±20% relative error bands around the reference value.

**Table S1:** Prompting environment and model identifiers used for the primary analysis. All models were accessed through their publicly available web interfaces without using APIs or a logged‐in personal account. No VPN or location‐masking tools were used.

**Table S2:** Description of each meal with expert and AI‐models estimation.

**Table S3:** Model performance according to clinically interpretable absolute‐error thresholds and carbohydrate‐load strata (< 20, 20–50, > 50 g).

**Table S4:** Clinical translation of carbohydrate‐counting error into theoretical and relative insulin‐dose differences overall and in meals containing > 50 g of carbohydrates.

**Table S5:** Errors ChatGPT table.

## Data Availability

All data supporting the findings of this study are available within the paper and its Supporting Information.
