# Supplementary figures and images for: Accuracy of ChatGPT, Gemini, Claude and DeepSeek in Carbohydrate Counting
Source: Diabetes Obes Metab. 2026 Apr 13;28(7):5627–36. doi: 10.1111/dom.70747 (PMC13243987; doi:10.1111/dom.70747)

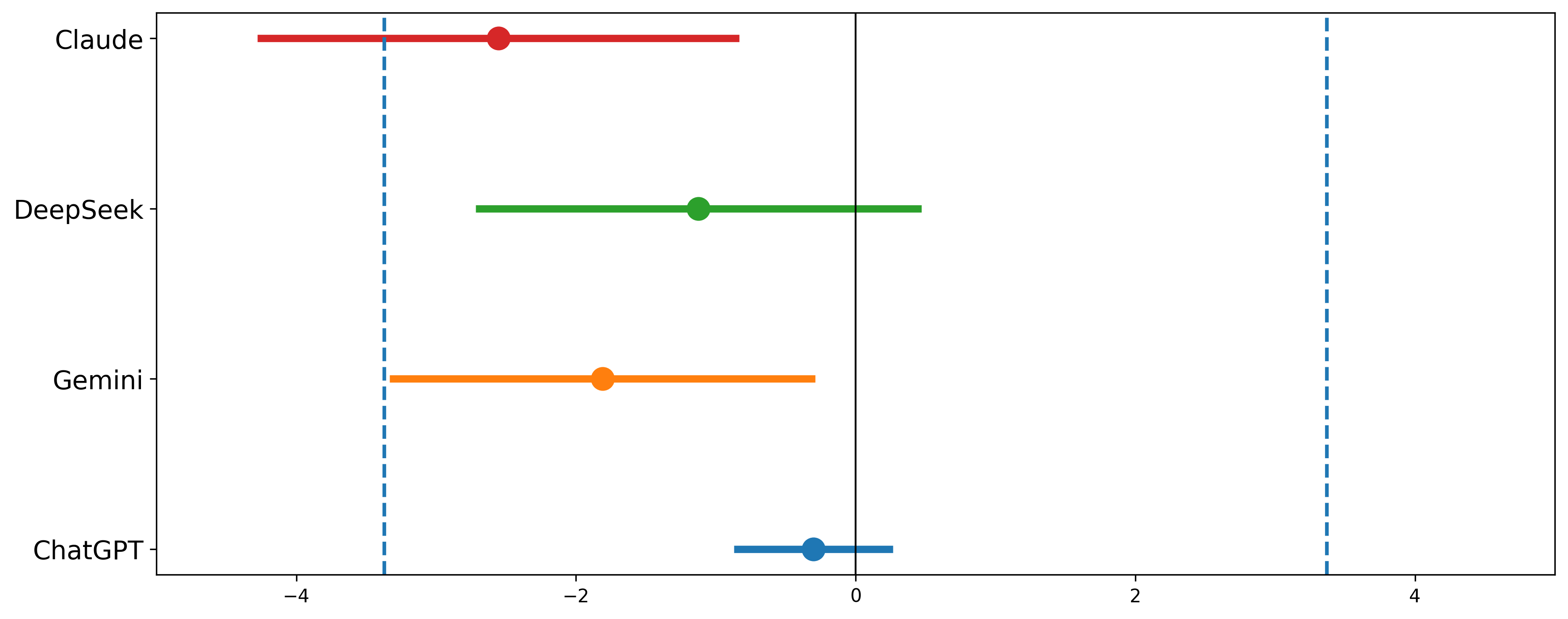

Supplement: Supplementary file 1 — Figure S1: Equivalence analysis of AI‐generated carbohydrate estimates compared with reference values. The x‐axis represents the mean difference (model − reference value, g) and the y‐axis the evaluated AI models; points indicate mean differences and horizontal lines 90% confidence intervals. Dashed vertical lines denote the equivalence margins (±10% of the mean reference value), used to explore potential statistical equivalence. [file DOM-28-5627-s007.png]

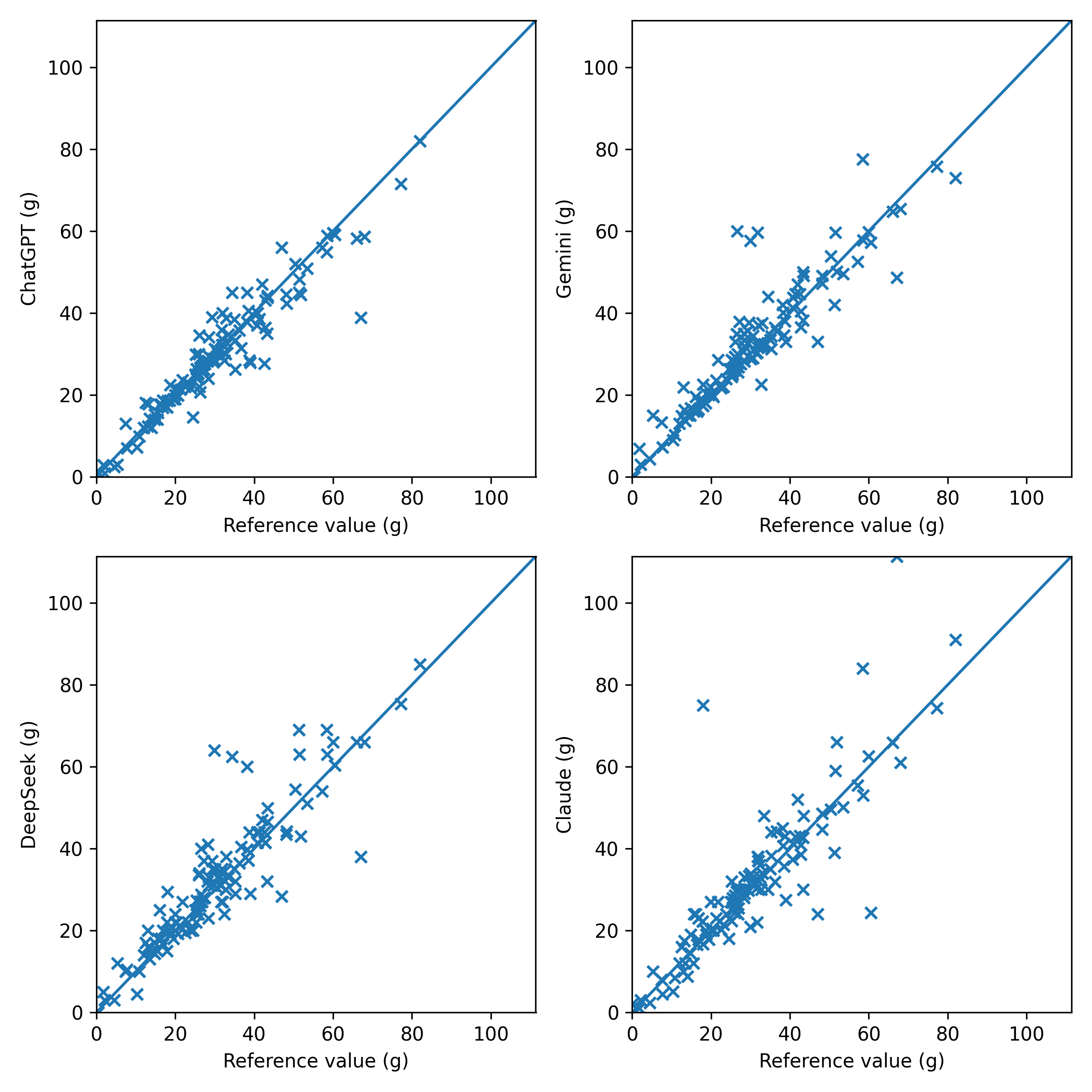

Supplement: Supplementary file 2 — Figure S2: Scatter plots comparing AI‐generated carbohydrate calculations with reference values calculated by clinicians. Each panel represents one AI model. The x‐axis shows the reference value (g) and the y‐axis the corresponding AI estimate (g), using the same scale across all panels. The diagonal line indicates the line of identity (y = x), representing perfect agreement. [file DOM-28-5627-s006.png]

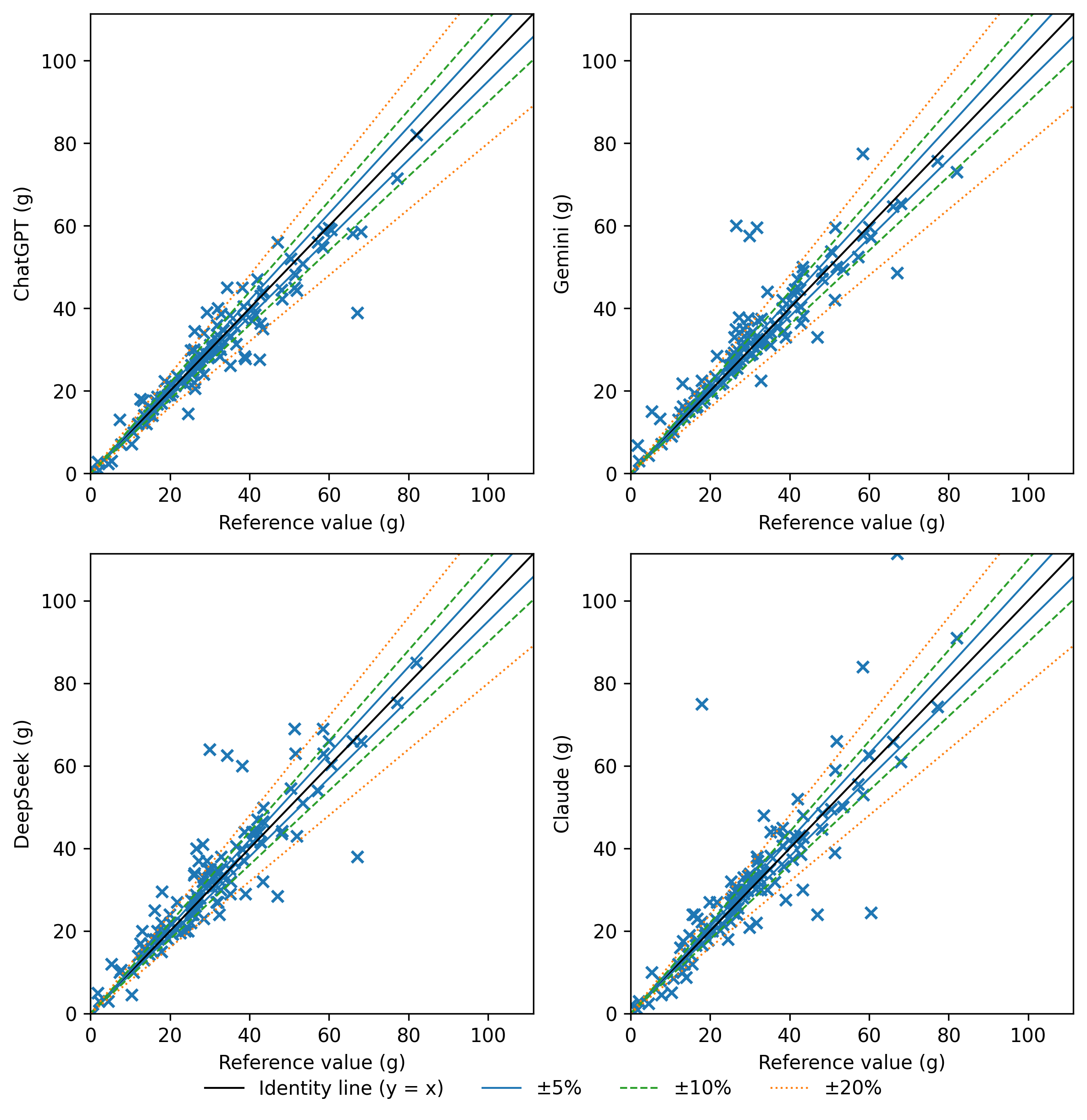

Supplement: Supplementary file 3 — Figure S3: Scatter plots of AI‐generated carbohydrate calculations versus clinicians' calculation values with relative error bands. The x‐axis represents the reference value (g) and the y‐axis the corresponding AI estimate (g), using the same scale across all panels. The solid diagonal line indicates the line of identity (y = x), while the additional diagonal lines represent ±5%, ±10% and ±20% relative error bands around the reference value. [file DOM-28-5627-s001.png]
