# Supplementary material for: Accuracy of ChatGPT, Gemini, Claude and DeepSeek in Carbohydrate Counting
Source: Diabetes Obes Metab. 2026 Apr 13;28(7):5627–36. doi: 10.1111/dom.70747 (PMC13243987; doi:10.1111/dom.70747)
Supplement: Supplementary file 4 — Table S1: Prompting environment and model identifiers used for the primary analysis. All models were accessed through their publicly available web interfaces without using APIs or a logged‐in personal account. No VPN or location‐masking tools were used. [file DOM-28-5627-s002.docx]

**Supplementary Table 1.** Prompting environment and model identifiers used for the primary analysis. All models were accessed through their publicly available web interfaces without using APIs or a logged-in personal account. No VPN or location-masking tools were used.

| **Platform** | **Model name as displayed** | **Access mode** | **Browser** | **Logged-in account** | **Country** | **Prompt language** | **Prompting period** | **Session isolation procedure** | **Output selection rule** |
| --- | --- | --- | --- | --- | --- | --- | --- | --- | --- |
| ChatGPT | ChatGPT-5.0 | Web-based interface | Google Chrome | No | Italy | English | July-September 2025; repeated in November 2025 after version update | Each meal entered in a new chat; conversation closed after each prompt; no follow-up prompts | When multiple outputs were available for the same meal, the output from the final prompting session using the most recent model version was retained |
| Gemini | Gemini 2.5 Flash | Web-based interface | Google Chrome | No | Italy | English | July-September 2025 | Each meal entered in a new chat; conversation closed after each prompt; no follow-up prompts | Single predefined output retained for statistical analysis |
| Claude | Claude 4 Sonnet | Web-based interface | Google Chrome | No | Italy | English | July-September 2025; repeated in November 2025 after version update | Each meal entered in a new chat; conversation closed after each prompt; no follow-up prompts | When multiple outputs were available for the same meal, the output from the final prompting session using the most recent model version was retained |
| DeepSeek | DeepSeek R1 | Web-based interface | Google Chrome | No | Italy | English | July-September 2025 | Each meal entered in a new chat; conversation closed after each prompt; no follow-up prompts | Single predefined output retained for statistical analysis |
