# Supplementary material for: Accuracy of ChatGPT, Gemini, Claude and DeepSeek in Carbohydrate Counting
Source: Diabetes Obes Metab. 2026 Apr 13;28(7):5627–36. doi: 10.1111/dom.70747 (PMC13243987; doi:10.1111/dom.70747)
Supplement: Supplementary file 5 — Table S2: Description of each meal with expert and AI‐models estimation. [file DOM-28-5627-s003.docx]

Supplementary table 2. Description of each meal with expert and AI-models estimation

| ID | MEAL | MEAL DESCRIPTION | FOOD ITEM 1 | CHO ESTIMATED BY AI (ITEM 1) | CHO ESTIMATED BY EXPERT (ITEM 1) | FOOD ITEM 2 | CHO ESTIMATED BY AI (ITEM 2) | CHO ESTIMATED BY EXPERT (ITEM 2) | FOOD ITEM 3 | CHO ESTIMATED BY AI (ITEM 3) | CHO ESTIMATED BY EXPERT (ITEM 3) | FOOD ITEM 4 | CHO ESTIMATED BY AI (ITEM 4) | CHO ESTIMATED BY EXPERT (ITEM 4) | TOTAL CHO ESTIMATED BY AI | TOTAL CHO ESTIMATED BY EXPERT |
| --- | --- | --- | --- | --- | --- | --- | --- | --- | --- | --- | --- | --- | --- | --- | --- | --- |
| 1 | Breakfast | 200 ml of semi-skimmed milk (Parmalat) + 3 rusks (7 g each) with 10 g apricot jam per slice | 200 ml of semi-skimmed milk | ChatGPT: 10 g Gemini: 9.6 g DeepSeek: 9.6 g Claude: 9.6 g | 9.8 g | 3 rusks (7 g each) | ChatGPT: 15.8 g Gemini: 15.12 g DeepSeek: 16 g Claude: 15.1 g | 15.75g | 10 g apricot jam per slice | ChatGPT: 19.5 g Gemini: 13.5 g DeepSeek: 21 g Claude: 18 g | 11.4 g |  |  |  | ChatGPT: 45.3 g Gemini: 38.2 g DeepSeek: 46.6 g Claude: 42.7 g | 36.94 g |
| 2 | Breakfast | 125 g sweetened whole plain yogurt (Müller) + 20 g oat flakes | 125 g sweetened whole plain yogurt | ChatGPT: 16.6g Gemini: 22.5 g DeepSeek: 18.75 g Claude: 17.5 g | 14.4 g | 20 g oat flakes | ChatGPT: 12.6 g Gemini: 12.4 g DeepSeek: 10 g Claude: 12 g | 13.36g |  |  |  |  |  |  | ChatGPT: 29.2 g Gemini: 34.9 g DeepSeek: 28.75 g Claude: 29.5 g | 27.76 g |
| 3 | Breakfast | 250 ml ACE juice in brick (Yoga) + 2 slices of wholemeal bread (30 g total). toasted | 250 ml ACE juice in brick (Yoga) | ChatGPT: 27.3 g Gemini: 27.5 g DeepSeek: 28.75 g Claude: 27.5 g | 25.25 g | 2 slices of wholemeal bread (30 g total). toasted | ChatGPT: 12.9 g Gemini: 12.6 g DeepSeek: 11 g Claude: 13.2 g | 13.23g |  |  |  |  |  |  | ChatGPT: 40.2 g Gemini: 40.1 g DeepSeek: 39.75 g Claude: 40.7 g | 38.48 g |
| 4 | Breakfast | 150 ml whole milk + 2 Oswego-type biscuits (10 g each) | 150 ml whole milk | ChatGPT: 7.3 g Gemini: 7.2 g DeepSeek: 7.5 g Claude: 7.2 g | 7.2 g | 2 Oswego-type biscuits (10 g each) | ChatGPT: 14.5g Gemini: 14.4 g DeepSeek: 12 g Claude: 13 g | 15 g |  |  |  |  |  |  | ChatGPT: 21.8 g Gemini: 21.6 g DeepSeek: 19.5g Claude: 20.2 g | 22.2 g |
| 5 | Breakfast | 50 g packaged fruit tart (Mulino Bianco) | 50 g packaged fruit tart (Mulino Bianco) | ChatGPT: 26.3 g Gemini: 32.5 g DeepSeek: 32.5 g Claude: 32 g | 32.15g |  |  |  |  |  |  |  |  |  | ChatGPT: 26.3 g Gemini: 32.5 g DeepSeek: 32.5 g Claude: 32 g | 32.15g |
| 6 | Breakfast | 200 ml sweetened tea with 2 teaspoons of sugar (10 g) + 4 rusks (28 g total) | 200 ml of tea | ChatGPT: 0 g Gemini: 0 g DeepSeek: 0 g Claude: 0 g | 0 g | 2 teaspoons of sugar (10 g) | ChatGPT: 10g Gemini: 10 g DeepSeek: 10 g Claude: 10 g | 10.45g | 4 rusks (28 g total) | ChatGPT: 21 g Gemini: 20.16 g DeepSeek: 21 g Claude: 20.2 g | 21g |  |  |  | ChatGPT: 31 g Gemini: 30.16 g DeepSeek: 31 g Claude: 30.2 g | 31.45 g |
| 7 | Breakfast | 200 ml whole milk + 2 wholemeal biscuits (12 g each) | 200 ml whole milk | ChatGPT: 9.6g Gemini: 9.6 g DeepSeek: 9.6 g Claude: 9.6 g | 9.6 g | 2 wholemeal biscuits (12 g each) | ChatGPT: 16.3g Gemini: 16.32 g DeepSeek: 14.4 g Claude: 15.6 g | 17 g |  |  |  |  |  |  | ChatGPT: 25.9g Gemini: 25.92 g DeepSeek: 24 g Claude: 25.2 g | 26.6 g |
| 8 | Breakfast | 1 apple (120 g) + 1 packet wholemeal crackers (25 g Misura) | 1 apple (120 g) | ChatGPT: 14.4 g Gemini: 15.6 g DeepSeek: 16 g Claude: 14.4 g | 13.32 g | 1 packet wholemeal crackers (25 g Misura) | ChatGPT: 17.5 g Gemini: 16.25 g DeepSeek: 15 g Claude: 15.8 g | 15.25g |  |  |  |  |  |  | ChatGPT: 31.9 g Gemini: 31.85 g DeepSeek: 31 g Claude: 30.2 g | 28.57g |
| 9 | Breakfast | 150 ml semi-skimmed milk + 3 tablespoons corn flakes (15 g total) | 150 ml semi-skimmed milk | ChatGPT: 7.5 g Gemini: 7.2 g DeepSeek: 7.2 g Claude: 7.2 g | 7.2g | 3 tablespoons corn flakes (15 g total) | ChatGPT: 12 g Gemini:12.3 g DeepSeek: 12 g Claude: 12.8 g | 13.11g |  |  |  |  |  |  | ChatGPT: 19.5 g Gemini: 19.5 g DeepSeek: 19.2 g Claude: 20 g | 20.31g |
| 10 | Breakfast | 1 banana (130 g) + 1 slice common bread (40 g) | 1 banana (130 g) | ChatGPT: 26 g Gemini: 29.9 g DeepSeek: 29.9 g Claude: 26 g | 22.62g | 1 slice common bread (40 g) | ChatGPT: 20 g Gemini: 19.2 g DeepSeek: 20 g Claude: 22 g | 23.8g |  | usda 19.7 g |  |  |  |  | ChatGPT: 46 g Gemini: 49.1 g DeepSeek: 49.9 g Claude: 48 g | 46.42g |
| 11 | Breakfast | 125 g whole peach yogurt (Müller) + 1 tablespoon honey (10 g) | 125 g whole peach yogurt (Müller) | ChatGPT: 17.5 g Gemini: 21.25 g DeepSeek: 18.8 g Claude: 16.3 g | 18g | 1 tablespoon honey (10 g) | ChatGPT: 10 g Gemini: 8 g DeepSeek: 8.2 g Claude: 8 g | 8.03g |  |  |  |  |  |  | ChatGPT: 27.5 g Gemini: 29.25 g DeepSeek:27 g Claude: 24.3 g | 26.03g |
| 12 | Breakfast | 250 ml fresh orange juice + 1 packet dry biscuits (30 g) | 250 ml fresh orange juice | ChatGPT: 25g Gemini: 27.5 g DeepSeek: 22.5 g Claude: 22.5 g | 20.5 g | 1 packet dry biscuits (30 g) | ChatGPT: 21.8 g Gemini: 21.6 g DeepSeek: 21 g Claude: 22.2 g | 24.12g |  |  |  |  |  |  | ChatGPT: 46.8 g Gemini: 49.1 g DeepSeek: 43.5 g Claude: 44.7 g | 44.62g |
| 13 | Breakfast | 1 slice wholemeal bread (40 g) with 15 g strawberry jam + 1 apple (100 g) | 1 slice wholemeal bread (40 g) | ChatGPT: 17.2 g Gemini: 16.8 g DeepSeek: 18 g Claude: 17.6 g | 17.64g | 15 g strawberry jam | ChatGPT: 9.3 g Gemini: 6.75 g DeepSeek: 10.5 g Claude: 9 g | 5.55 g | 1 apple (100 g) | ChatGPT: 12 g Gemini: 13 g DeepSeek: 13 g Claude: 12 g | 11.1 g |  |  |  | ChatGPT: 38.5 g Gemini: 36.55 g DeepSeek: 41.5 g Claude: 38.6 g | 34.29g |
| 14 | Breakfast | 150 g whole plain yogurt + 30 g whole muesli | 150 g whole plain yogurt | ChatGPT: 18.8 g Gemini: 7.2 g DeepSeek: 7.5 g Claude: 6.9 g | 6.45g | 30 g whole muesli | ChatGPT: 18.8 g Gemini: 19.5 g DeepSeek: 20.4 g Claude: 18.6 g | 21.51g |  |  |  |  |  |  | ChatGPT: 37.6 g Gemini: 26.7 g DeepSeek: 27.9 g Claude: 25.5 g | 27.96g |
| 15 | Breakfast | 200 ml milk with 10 g Nesquik + 1 butter cookie (15 g) | 200 ml of milk | ChatGPT: 10g Gemini: 9.6 g DeepSeek: 9.4 g Claude: 9.6 g | 9.8g | 10 g Nesquik | ChatGPT: 8 g Gemini: 8.2 g DeepSeek: 8.5 g Claude: 8.5 g | 7.89g | 1 butter cookie (15 g) | ChatGPT: 10 g Gemini: 10.5 g DeepSeek: 9.5 g Claude: 9.8 g | 9.6 g |  |  |  | ChatGPT: 28g Gemini: 28.3 g DeepSeek: 27.4 g Claude: 27.9 g | 27.29g |
| 16 | Breakfast | 200 ml instant barley coffee (prepared with water and 1 teaspoon sugar) + 2 rusks (14 g total | 200 ml instant barley coffee (prepared with water and 1 teaspoon sugar) | ChatGPT:11 g Gemini: 7.25 g DeepSeek: 19 g Claude: 6.6 g | 7.5g | 2 rusks (14 g total) | ChatGPT: 10.5 g Gemini: 10.08 g DeepSeek: 10.5 g Claude: g | 10.5 g |  |  |  |  |  |  | ChatGPT: 21.5 g Gemini: 17.3 g DeepSeek: 29.5 g Claude: 16.7 g | 18g |
| 17 | Breakfast | 200 ml soy milk (Alpro) + 20 g whole cereals | 200 ml soy milk (Alpro) | ChatGPT: 1.4 g Gemini: 7 g DeepSeek: 4 g Claude: 4.6 g | 5g | 20 g whole cereals | ChatGPT: 14g Gemini: 13 g DeepSeek: 14 g Claude: 14.2 g | 1.7g |  |  |  |  |  |  | ChatGPT: 15.4g Gemini: 20 g DeepSeek: 18 g Claude: 17.8 g | 6.7g |
| 18 | Breakfast | 200 ml tropical juice + 1 slice bread (50 g) with 10 g jam | 200 ml tropical juice | ChatGPT: 24g Gemini: 24 g DeepSeek: 22 g Claude: 22 g | 21.4g | 1 slice bread (50 g) | ChatGPT: 25 g Gemini: 24 g DeepSeek: 25 g Claude: 27.5 g | 29.75g | 10 g jam | ChatGPT: 5.5 g Gemini: 4.5 g DeepSeek: 7 g Claude: 6 g | 5.87g |  |  |  | ChatGPT: 54.5 g Gemini: 52.5 g DeepSeek: 54 g Claude: 55.5 g | 57.02g |
| 19 | Breakfast | 1 whole milk yogurt (125 g) strawberry flavor with 20 g corn flakes | 1 whole milk strawberry yogurt (125 g). Yomo | ChatGPT: 16.3 g Gemini: 21.25 g DeepSeek: 15 g Claude: 16.3 g | 16.2 g | 20 g corn flakes | ChatGPT: 16 g Gemini: 16.4 g DeepSeek: 16 g Claude: 17 g | 17.4g |  |  |  |  |  |  | ChatGPT: 32.3 g Gemini: 37.65 g DeepSeek: 31 g Claude: 33.3 g | 33.6 g |
| 20 | Breakfast | 1 glass semi-skimmed milk (100 ml) + 1 large wholemeal biscuit Misura (20 g) | 1 glass semi-skimmed milk (100 ml) | ChatGPT: 5g Gemini: 4.8 g DeepSeek: 4.7 g Claude: 4.8 g | 4.9g | 1 large wholemeal biscuit Misura (20 g) | ChatGPT: 13.5 g Gemini: 13.6 g DeepSeek: 12 g Claude: 13 g | 11.8g |  |  |  |  |  |  | ChatGPT: 18.5 g Gemini: 18.4 g DeepSeek: 16.7 g Claude: 17.8 g | 16.7g |
| 21 | Breakfast | 1 banana (140 g) + 3 classic rusks (30 g) | 1 banana 140 g | ChatGPT: 28 g Gemini: 32.2 g DeepSeek: 32 g Claude: 28 g | 24.36g | 3 classic rusks (30 g) | ChatGPT: 22.5 g Gemini: 21.6 g DeepSeek: 22.5 g Claude: 21.6 g | 22.5g |  |  |  |  |  |  | ChatGPT: 50.5 g Gemini: 53.8 g DeepSeek: 54.5 g Claude: 49.6 g | 46.86g |
| 22 | Breakfast | 150 ml whole milk with 15 g cocoa powder + 2 wholemeal rusks (20 g) | 150 ml whole milk | ChatGPT: 7.3g Gemini: 7.2 g DeepSeek: 7 g Claude: 7.2 g | 7.35g | 15 g cocoa powder | ChatGPT: 1.5g Gemini: 1.8 g DeepSeek: 3 g Claude: 1.7 g | 1.73g | 2 wholemeal rusks (20 g) | ChatGPT: 14 g Gemini: 13 g DeepSeek: 12 g Claude: 12.6 g | 12.6g |  |  |  | ChatGPT: 22.8 g Gemini: 22 g DeepSeek: 22 g Claude: 21.5 g | 21.68g |
| 23 | Breakfast | 200 ml orange juice + 1 slice wholemeal bread (50 g) with 30 g cream cheese (Certosa) | 200 ml orange juice | ChatGPT: 20 g Gemini: 22 g DeepSeek: 18 g Claude: 18 g | 16.4g | 1 slice wholemeal bread (50 g) | ChatGPT: 22.5 g Gemini: 21 g DeepSeek: 22 g Claude: 22 g | 22.05g | 30 g cream cheese (Certosa) | ChatGPT: 0 g Gemini: 0.75 g DeepSeek: 1.5 g Claude: 1.2 g | 0.75g |  |  |  | ChatGPT: 42.8 g Gemini: 43.75 g DeepSeek: 41.5 g Claude: 41.2 g | 39.2g |
| 24 | Breakfast | 1 low-fat Greek yogurt (150 g) with 10 g honey + 20 g muesli | 1 low-fat Greek yogurt (150 g) | ChatGPT: 6 g Gemini: 9 g DeepSeek: 5.3 g Claude: 6 g | 6g | 10 g honey | ChatGPT: 8 g Gemini: 8 g DeepSeek: 8.2 g Claude: 8 g | 8g | 20 g muesli | ChatGPT: 12.5 g Gemini: 13 g DeepSeek: 13.6 g Claude: 12.4 g | 14.3g |  |  |  | ChatGPT: 26.5 g Gemini: 30 g DeepSeek: 27.1 g Claude: 26.4 g | 28.3g |
| 25 | Breakfast | 1 pear (100 g) with 2 slices Parma ham (25 g) | 1 pear 100 g | ChatGPT: 10.5 g Gemini: 15 g DeepSeek: 15g Claude: 8.8 g | 10.2g | 2 slices Parma ham (25 g) | ChatGPT: 0g Gemini: 0 g DeepSeek: 0 g Claude: 0 g | 0g |  |  |  |  |  |  | ChatGPT: 10.5g Gemini: 15 g DeepSeek: 15 g Claude: 8.8 g | 10.2g |
| 26 | Breakfast | 2 pork sausages (50 g) + 2 boiled eggs | 2 pork sausages (50 g) | ChatGPT: 0.5g Gemini: 0 g DeepSeek: 0 g Claude: 0 g | 0g | 2 boiled eggs | ChatGPT: 0 g Gemini: 0 g DeepSeek: 0 g Claude: 0 g | 0g |  |  |  |  |  |  | ChatGPT: 0.5 g Gemini: 0 g DeepSeek: 0 g Claude: 0 g | 0g |
| 27 | Breakfast | 1 cup green tea (150 ml) unsweetened with 1 Pavesi Gocciola cookie | 1 cup green tea (150 ml) unsweetened | ChatGPT: 0g Gemini: 0 g DeepSeek: 0 g Claude: 0 g | 0g | 1 Pavesi Gocciola cookie | ChatGPT: 8.4 g Gemini: 7.2 g DeepSeek: 10.5 g Claude: 4.5 g | 7.7g |  |  |  |  |  |  | ChatGPT: 8.4 g Gemini: 7.2 g DeepSeek: 10.5 g Claude: 4.5 g | 7.7g |
| 28 | Breakfast | 200 ml rice milk + 1 plumcake (Bauli) 35 g | 200 ml rice milk unsweetened | ChatGPT: 6.4 g Gemini: 22 g DeepSeek: 24 g Claude: 18 g | 7.4 g | 1 plumcake (Bauli) 35 g | ChatGPT: 14 g Gemini: 19.25 g DeepSeek: 20 g Claude: 19.3 g | 14.7g |  |  |  |  |  |  | ChatGPT: 20.4 g Gemini: 41.25 g DeepSeek: 44 g Claude: 37.3 g | 22.1g |
| 29 | Breakfast | 1 wholemeal sandwich (75 g) with 30 g stracchino cheese | 1 wholemeal sandwich (75 g) | ChatGPT: 32.3g Gemini: 31.5vg DeepSeek: 33 g Claude: 33 g | 33g | 30 g stracchino cheese | ChatGPT: 0.5g Gemini: 0.45 g DeepSeek: 0.3 g Claude: 0.9 g | 0g |  |  |  |  |  |  | ChatGPT: 32.8g Gemini: 31.95 g DeepSeek: 33.3 g Claude: 33.9 g | 33g |
| 30 | Breakfast | 1 whole banana yogurt (125 g) + grilled bacon (100 g) | 1 whole banana yogurt (125 g) yomo | ChatGPT: 16.4g Gemini: 0 g DeepSeek: 0 g Claude: 0 g | 16.2g | grilled bacon (100 g) | ChatGPT: 0g Gemini: 0 g DeepSeek: 0 g Claude: 0 g | 0g |  |  |  |  |  |  | ChatGPT: 16.4g Gemini:15 g DeepSeek: 14.3 g Claude: 14.5 g | 16.2g |
| 31 | Breakfast | 150 ml lactose-free milk (Zymil) with 3 slices wholemeal bread (60 g) with Nutella (Ferrero) 30 g | 150 ml lactose-free milk (Zymil) | ChatGPT: 7.5 g Gemini: 7.2 g DeepSeek: 7.5 g Claude: 7.2 g | 7.35g | 3 slices wholemeal bread (60 g) | ChatGPT: 25.8 g Gemini: 25.2 g DeepSeek: 26.4 g Claude: 26.4 g | 26.46 g | Nutella (Ferrero) 30 g | ChatGPT: 17.3g Gemini: 17.1 g DeepSeek: 17.1 g Claude: 16.5 g | 17.25g |  |  |  | ChatGPT: 50.6g Gemini: 49.5 g DeepSeek: 51 g Claude: 50.1 g | 51.6g |
| 32 | Lunch | 80 g durum wheat pasta with tomato sauce (60 g) + 5 g EVO oil and 10 g grated parmesan | 80 g pasta | ChatGPT: 56.8 g Gemini: 57.6 g DeepSeek: 62.4 g Claude: 58.4 g | 58.2 g | tomato sauce (60 g) | ChatGPT: 2.7g Gemini: 2.1 g DeepSeek: 3.6 g Claude: 4.2 g | 2.16g | 5 g EVO oil | ChatGPT: 0 g Gemini: 0 g DeepSeek: 0 g Claude: 0 g | 0 g | 10 g grated parmesan | ChatGPT: 0.09 g Gemini: 0 g DeepSeek: 0 g Claude: 0 g | 0 g | ChatGPT: 59.59g Gemini: 59.7 g DeepSeek: 66 g Claude: 62.6 g | 60.36 g |
| 33 | Lunch | 150 g potatoes + 100 g grilled chicken breast + 5 g EVO oil | 150 g potatoes | ChatGPT: 25.5g Gemini: 25.5 g DeepSeek: 24 g Claude: 26.9 g | 24 g | 100 g grilled chicken breast | ChatGPT: 0 g Gemini: 0 g DeepSeek: 0 g Claude: 0 g | 0 g | 5 g EVO oil | ChatGPT: 0 g Gemini: 0 g DeepSeek: 0 g Claude: 0 g | 0 g |  |  |  | ChatGPT: 25.5 g Gemini: 25.5 g DeepSeek: 24 g Claude: 26.9 g | 24 g |
| 34 | Lunch | 2 boiled eggs (100 g) + 40 g bread + 150 g raw carrots | 2 boiled eggs (100 g) | ChatGPT: 0.5 g Gemini: 0 g DeepSeek: 0 g Claude: 0 g | 0 g | 40 g bread | ChatGPT: 22 g Gemini: 19.2 g DeepSeek: 20 g Claude: 23.8 g | 23.8 g | 150 g raw carrots | ChatGPT: 10.5 g Gemini: 12 g DeepSeek: 9 g Claude: 14.4 g | 11.4 g |  |  |  | ChatGPT: 33.1g Gemini: 31.2 g DeepSeek: 29 g Claude: 38.2 g | 35.2 g |
| 35 | Lunch | 150 g mixed salad + 50 g wholemeal bread + 1 apple (120 g) | 150 g mixed salad | ChatGPT: 2.25 g Gemini: 3.75 g DeepSeek: 3 g Claude: 4.5 g | 3.3 g | 50 g wholemeal bread | ChatGPT: 22 g Gemini: 21 g DeepSeek: 22.5 g Claude: 22.1 g | 22.05 g | 1 apple (120 g) (Golden) | ChatGPT: 14.4 g Gemini: 15.6 g DeepSeek: 16 g Claude: 14.3 g | 12.84g |  |  |  | ChatGPT: 38.7 g Gemini: 40.35 g DeepSeek: 41.5 g Claude: 40.9 g | 38.19 g |
| 36 | Lunch | 70 g polished rice. plain. with 1 tablespoon EVO oil (12 g) + 1 apple (150 g) | 70 g polished rice. plain. | ChatGPT: 54.6 g Gemini: 56.2 g DeepSeek: 55.3 g Claude: 56.3 g | 56.2 g | 1 tablespoon EVO oil (12 g) | ChatGPT: 0 g Gemini: 0 g DeepSeek: 0 g Claude: 0 g | 0 g | 1 apple (150 g) (Golden) | ChatGPT: 18 g Gemini: 19.5 g DeepSeek: 20 g Claude: 18 g | 16.1 g |  |  |  | ChatGPT: 72.6. Gemini: 75.7 g DeepSeek: 75.3 g Claude: 74.3 g | 72.3g |
| 37 | Lunch | 90 g egg pasta with sautéed zucchini (100 g) + 10 g EVO oil | 90 g egg pasta | ChatGPT: 58.5 g Gemini: 61.2 g DeepSeek: 63 g Claude: 63.9 g | 63.9 g | sautéed zucchini (100 g) | ChatGPT: 2 g Gemini: 3.5 g DeepSeek: 3 g Claude: 2 g | 2.9 g | 10 g EVO oil | ChatGPT: 0 g Gemini: 0 g DeepSeek: 0 g Claude: 0 g | 0 g |  |  |  | ChatGPT: 60.8 g Gemini: 64.7 g DeepSeek: 66 g Claude: 65.9 g | 66.8 g |
| 38 | Lunch | 100 g potato gnocchi with buffalo mozzarella (70 g) and tomato sauce (80 g) + 5 g parmesan | 100 g potato gnocchi | ChatGPT: 30 g Gemini: 32 g DeepSeek: 35 g Claude: 37.9 g | 34 g | buffalo mozzarella (70 g) | ChatGPT: 0.7 g Gemini: 0.7 g DeepSeek: 0.7 g Claude: 0.7 g | 0.98 g | tomato sauce (80 g) | ChatGPT: 3.6 g Gemini: 2.8 g DeepSeek: 4.8 g Claude: 5.6 g | 2.9g | 5 g parmesan | ChatGPT: 0 g Gemini: 0 g DeepSeek: 0 g Claude: 0 g | 0 g | ChatGPT: 34.3 g Gemini: 35.5 g DeepSeek: 40.5 g Claude: 44.2 g | 37.88 g |
| 39 | Lunch | 1 beef burger (100 g) + 60 g rye bread + 100 g tomatoes | 1 beef burger (100 g) | ChatGPT: 0 g Gemini: 0 g DeepSeek: 0 g Claude: 0 g | 0 g | 60 g rye bread | ChatGPT: 27 g Gemini: 25.2 g DeepSeek: 27 g Claude: 30 g | 27.24 g | 100 g tomatoes | ChatGPT: 3.5g Gemini: 3.5 g DeepSeek: 3 g Claude: 3.9 g | 3.5 g |  |  |  | ChatGPT: 30.5 g Gemini: 28.7 g DeepSeek: 35 g Claude: 33.9 g | 30.74 g |
| 40 | Lunch | 150 g grilled turkey + 50 g wholemeal bread + 1 orange (80 g) | 150 g grilled turkey | ChatGPT: 0 g Gemini: 0 g DeepSeek: 0 g Claude: 0 g | 0 g | 50 g wholemeal bread | ChatGPT: 21 g Gemini: 21 g DeepSeek: 22.5 g Claude: 22 g | 22.05 g | 1 orange (80 g) without peel | ChatGPT: 7.2 g Gemini: 8 g DeepSeek: 8 g Claude: 9.6 g | 6.24 g |  |  |  | ChatGPT: 28.2 g Gemini: 29 g DeepSeek: 30.5 g Claude: 31.6 g | 28.3 g |
| 41 | Lunch | 70 g basmati rice + 100 g carrots + 5 g EVO oil | 70 g basmati rice | ChatGPT: 53.9g Gemini: 19.6 g DeepSeek: 18 g Claude: 20.3 g | 58.03 g | 100 g carrots ( ) | ChatGPT: 7 g Gemini: 8 g DeepSeek: 8 g Claude: 7.4 g | 7.6 g | 5 g EVO oil | ChatGPT: 0 g Gemini: 0 g DeepSeek: 0 g Claude: 0 g |  |  |  |  | ChatGPT: 61 g Gemini: 27.6 g DeepSeek: 26 g Claude: 27.7 g | 65.6 g |
| 42 | Lunch | 80 g dry pasta with butter and parmesan (10 g butter + 10 g parmesan) | 80 g di pasta secca | ChatGPT:60g Gemini: 57.6 g DeepSeek: 64 g Claude: 20.9 g | 58.16 g | 10 g butter | ChatGPT: 0.1 g Gemini: 0 g DeepSeek: 0.1 g Claude: 0.1 g | 0 g | 10 g parmesan | ChatGPT: 0.09 g Gemini: 0 g DeepSeek: 0 g Claude: 0 g | 0 |  |  |  | ChatGPT: 60.1g Gemini: 57.6 g DeepSeek: 64 g Claude: 20.9 g | 58.26 g |
| 43 | Lunch | 250 ml vegetable broth with 30 g semolina pasta + 1 banana (100 g) | 250 ml vegetable broth | ChatGPT: 2g Gemini: 0 g DeepSeek: 0 g Claude: 1.3 g | 0 g | 30 g semolina pasta | ChatGPT: 22.5 g Gemini: 21.6 g DeepSeek: 24 g Claude: 21.6 g | 21.81 g | 1 banana 100 g | ChatGPT: 22g Gemini: 23 g DeepSeek: 20 g Claude: 20 g | 17.4 g |  |  |  | ChatGPT: 46.5 g Gemini: 44.6 g DeepSeek: 44 g Claude: 42.9 g | 39.21 g |
| 44 | Lunch | 1 Happy Meal from McDonald’s: 1 cheeseburger + 1 portion of French fries + 1 classic Coca-Cola | 1 cheeseburger | ChatGPT: 30 g Gemini: 31 g DeepSeek: 31 g Claude: 31 g | 30 g | 1 portion of French fries | ChatGPT: 29.9 g Gemini: 17 g DeepSeek: 28 g Claude: 25 g | 29 g | 1 classic Coca-Cola (0.33L) | ChatGPT: 26.5 g Gemini: 25 g DeepSeek: 26 g Claude: 35 g | 35 g (lattina coca cola da 0.33L) | |  |  | ChatGPT: 86.4g Gemini: 73 g DeepSeek: 85 g Claude: 91 g | 94 g |
| 45 | Lunch | 2 beef meatballs (100 g) + 150 g of baked potatoes + 5 g of extra virgin olive oil | 2 beef meatballs (100 g) | ChatGPT:3 g Gemini: 7.5 g DeepSeek: 5 g Claude: 2 g | 5.9 g (10 g pane) | 150 g of baked potatoes | ChatGPT: 32.5 g Gemini: 25.5 g DeepSeek: 24 g Claude: 25.5 g | 38.55 g | 5 g EVO oil | ChatGPT: 0 g Gemini: 0 g DeepSeek: 0 g Claude: 0 g | 0 g |  |  |  | ChatGPT: 35.5 g Gemini: 33 g DeepSeek: 29 g Claude: 27.5 g | 30.9 g (44.45 gr) |
| 46 | Lunch | Big salad with 80 g canned tuna. 30 g corn. 150 g lettuce + 50 g crackers (Pavesi) | 80 g canned tuna | ChatGPT: 0 g Gemini: 0 g DeepSeek: 0 g Claude: 0 g | 0 g | 30 g corn | ChatGPT: 5.7 g Gemini: 5.1 g DeepSeek: 6 g Claude: 6.6 g | 5.9 g | 150 g lettuce | ChatGPT: 3 g Gemini: 3 g DeepSeek: 3 g Claude: 5 g | 4.5 g | 50 g crackers (Pavesi) | ChatGPT: 35g Gemini: 36.5 g DeepSeek: 35 g Claude: 35 g | 33.1 g | ChatGPT: 43.7g Gemini: 44.6 g DeepSeek: 44 g Claude: 43.1 g | 43.5 g |
| 47 | Lunch | Toast with 2 slices sandwich bread (50 g) with 50 g cooked ham + 30 g cream cheese (Philadelphia) | Toast with 2 slices sandwich bread (50 g) | ChatGPT: 25g Gemini: 24 g DeepSeek: 25 g Claude: 24 g | 24 g | 50 g cooked ham | ChatGPT: 0 g Gemini: 0 g DeepSeek: 0.5 g Claude: 0.5 g | 0.8 g | 30 g cream cheese (Philadelphia) | ChatGPT: 0.9 g Gemini: 1.05 g DeepSeek: 1 g Claude: 1.2 g | 1 g | 1 fetta di melone retato (90 g) | ChatGPT: 6.3g Gemini: 6.75 g DeepSeek: 8 g Claude: 7.2 g | 6.67 g | ChatGPT: 32.2 g Gemini: 31.8 g DeepSeek: 34.5 g Claude: 32.9 g | 32.47 g |
| 48 | Lunch | 80 g cold pasta with 40 g mozzarella (Vallelata) and 40 g cherry tomatoes + 1 peach (120 g) | 80 g cold pasta | ChatGPT: 56 g Gemini: 23.2 g DeepSeek: 22 g Claude: 20.8 g | 56.8 g | 40 g mozzarella (Vallelata) | ChatGPT: 0.5g Gemini: 0.4 g DeepSeek: 1 g Claude: 1.2 g | 0.4 g | 40 g cherry tomatoes | ChatGPT: 1.2 g Gemini: 1.4 g DeepSeek: 1.5 g Claude: 1.6 g | 1.6 g | 1 peach (120 g) | ChatGPT: 12g Gemini: 11.4 g DeepSeek: 12 g Claude: 8.4 g | 11.4 g | ChatGPT: 69.7 g Gemini: 36.4g DeepSeek: 36.5 g Claude: 31.9 g | 70.2 g |
| 49 | Lunch | 70 g black rice with 100 g canned lentils + 80 g canned peas | 70 g black rice | ChatGPT: 52.5 g Gemini: 19.6 g DeepSeek: 21 g Claude: 18.2 g | 56.3 g | 100 g canned lentils | ChatGPT: 12 g Gemini: 18 g DeepSeek: 20 g Claude: 20 g | 15.4 g | 80 g canned peas | ChatGPT: 8 g Gemini: 9.6 g DeepSeek: 11.2 g Claude: 10.4 g | 8.9 g |  |  |  | ChatGPT: 72.5 g Gemini: 47.2 g DeepSeek: 44.2 g Claude: 48.6 g | 82.6 g |
| 50 | Lunch | 80 g rigatoni (Barilla) with 30 g pesto | 80 g rigatoni (Barilla) | ChatGPT: 60 g Gemini: 23.2 g DeepSeek: 24 g Claude: 20.8 g | 56.9 g | 30 g pesto | ChatGPT: 1.5g Gemini: 1.2 g DeepSeek: 0.9 g Claude: 1.5 g | 3.3 g PESTO BARILLA | |  |  |  |  |  | ChatGPT: 61.5 g Gemini: 24.4 g DeepSeek: 24.9 g Claude: 22.3 g | 60.2 g |
| 51 | Lunch | 100 g Basmati risotto with mixed mushrooms (130 g) | 100 g Basmati risotto | ChatGPT: 77 g Gemini: 28 g DeepSeek: 31.2 g Claude: 29 g | 77 g BASMATI GALLO | mixed mushrooms (130 g) | ChatGPT: 3.9 g Gemini: 4.55 g DeepSeek: 3.9 g Claude: 0.8 g | 2 g MISTO FUNGHI SUGELATO CONAD | |  |  |  |  |  | ChatGPT: 80.9 g Gemini: 32.55 g DeepSeek: 35 g Claude: 29.8 g | 79 g |
| 52 | Lunch | Chicken burger with “Bun” bread (60 g) + 20 g ketchup | Chicken burger | ChatGPT: 0 g Gemini: 0 g DeepSeek: 0 g Claude: 0 g | 0 g | “Bun” bread (60 g) COOP | ChatGPT: 30g Gemini: 28.8 g DeepSeek: 30 g Claude: 30 g | 30 g | 20 g Ketchup | ChatGPT: 5 g Gemini: 4.6 g DeepSeek: 5 g Claude: 5 g | 4.8 g senna |  |  |  | ChatGPT: 35 g Gemini: 33.4 g DeepSeek: 35 g Claude: 35 g | 34.8 g |
| 53 | Lunch | Meat cannelloni 250 g | Meat cannelloni 250 g | ChatGPT: 45 g Gemini: 50 g DeepSeek: 32 g Claude: 30 g | 25 g Cucina esselunga | |  |  |  |  |  |  |  |  | ChatGPT: 45 g Gemini: 50 g DeepSeek: 32 g Claude: 30 g | 25 g |
| 54 | Lunch | 80 g dry egg pasta Bolognese with 70 g ground veal | 80 g dry egg pasta | ChatGPT: 56 g Gemini: 54.4 g DeepSeek: 56 g Claude: 21.6 g | 56.8 g | 70 g ground veal | ChatGPT: 0 g Gemini: 2.8 g DeepSeek: 4.3 g Claude: 2.8 g |  |  |  |  |  |  |  | ChatGPT: 56 g Gemini: 57.2 g DeepSeek: 60.3 g Claude: 24.4 g | 56.8 g |
| 55 | Lunch | 150 g margherita pizza | 150 g pizza margherita | ChatGPT: 42g Gemini: 42 g DeepSeek: 69 g Claude: 39g | 41.1 g |  |  |  |  |  |  |  |  |  | ChatGPT: 42 g Gemini: 42 g DeepSeek: 69 g Claude: 39g | 41.1 g |
| 56 | Lunch | 200 g lasagna with meat sauce | 200 g lasagna | ChatGPT: 26 g Gemini: 44 g DeepSeek: 62.5 g Claude: 30g | 26.8 g( crea) |  |  |  |  |  |  |  |  |  | ChatGPT: 26 g Gemini: 44 g DeepSeek: 62.5 g Claude: 30g | 26.8 g |
| 57 | Lunch | Sandwich with 80 g wholemeal bread + 50 g cooked ham + 30 g galbanino cheese (Galbani) | Sandwich with 80 g wholemeal bread | ChatGPT: 36 g Gemini: 33.6 g DeepSeek: 36 g Claude: 35.2g | 37.6 g | 50 g cooked ham | ChatGPT: 0 g Gemini: 0 g DeepSeek: 0.5 g Claude: 0.5 g | 0.4 g | 30 g galbanino cheese (Galbani) | ChatGPT: 0.3g Gemini: 0.75 g DeepSeek: 0.6 g Claude: 0 g | 0.4 g |  |  |  | ChatGPT: 36.3 g Gemini: 34.5 g DeepSeek: 37.1 g Claude: 35.7g | 38.4 g |
| 58 | Lunch | 80 g couscous with 100 g grilled spinach + 20 g chickpeas | 80 g couscous | ChatGPT: 56g Gemini: 58.4 g DeepSeek: 60 g Claude: 56 g | 54.4 de cecco | 100 g grilled spinach | ChatGPT: 3.6 g Gemini: 3 g DeepSeek: 3 g Claude: 1.4 g | 3.6 g | 20 g chickpeas | ChatGPT: 3g Gemini: 4 g DeepSeek: 3 g Claude: 3.6g | 2.4 g |  |  |  | ChatGPT: 62.6 g Gemini: 65.4 g DeepSeek: 66 g Claude: 61g | 60.4 g |
| 59 | Lunch | 150 g baked pasta (cooked? 65 g raw?) | 150 g baked pasta (cooked? 65 g raw?) | ChatGPT: 60 g Gemini: 33 g DeepSeek: 28.4 g Claude: 24 g | 47 g |  |  |  |  |  |  |  |  |  | ChatGPT: 56 g Gemini: 33 g DeepSeek: 28.4 g Claude: 24 g | 47 g |
| 60 | Lunch | 100 g grilled beef + 150 g Brussels sprouts | 100 g grilled beef | ChatGPT: 0 g Gemini: 0 g DeepSeek: 0 g Claude: 0 g | 0 g | 150 g Brussels sprouts | ChatGPT: 8.5g Gemini: 9 g DeepSeek: 4.5 g Claude: 5.1 g | 6.3 g |  |  |  |  |  |  | ChatGPT: 8.5g Gemini: 9 g DeepSeek: 4.5 g Claude: 5.1 g | 6.3 g |
| 61 | Lunch | 75 g basmati rice with 80 g chicken curry | 75 g basmati rice | ChatGPT: 57.75 g Gemini: 21 g DeepSeek: 22 g Claude: 21.8 g | 57.8 gr basmati gallo | 80 g chicken curry | ChatGPT: 0 g Gemini: 4 g DeepSeek: 3 g Claude: 3.2 g | 0 se non c'è farina |  |  |  |  |  |  | ChatGPT: 57.75 g Gemini: 25 g DeepSeek: 25 g Claude: 25 g | 57.8 g Gallo |
| 62 | Lunch | 100 g ravioli with ricotta and spinach (Rana) with 60 g butter | 100 g ravioli with ricotta and spinach (Rana) | ChatGPT: 31g Gemini: 28 g DeepSeek: 33 g Claude: 28 g | 31 g | 60 g butter | ChatGPT: 0 g Gemini: 0 g DeepSeek: 0.6 g Claude: 0.6 g |  | 0 g |  |  |  |  |  | ChatGPT: 31 g Gemini: 28 g DeepSeek: 33.6 g Claude: 28.6 g | 31 g |
| 63 | Dinner | 200 g chickpea soup (100 g chickpeas) with 50 g white bread and 100 g apple with skin | 200 g chickpea soup (100 g chickpeas) CECI CRUDI | ChatGPT: 22g Gemini: 18.9 g DeepSeek: 27 g Claude: 27g | 14.80 g | 50 g white bread | ChatGPT: 25 g Gemini: 29.75 g DeepSeek: 25g Claude: 40g | 29.8 g | 100 g apple with skin | ChatGPT: 12g Gemini: 10 g DeepSeek: 14 g Claude: 14g | 12.00 g | quale mela? GOLDEN |  |  | ChatGPT: 59g Gemini: 58.6 g DeepSeek: 66 g Claude: 81g | 56.6 g |
| 64 | Dinner | 60 g pan bauletto bread (Mulino Bianco) with 50 g cooked ham and 100 g spinach | 60 g pan bauletto bread (Mulino Bianco) | ChatGPT: 29 g Gemini: 28.8 g DeepSeek: 30g Claude:45 g | 29.04 g | 50 g cooked ham | ChatGPT: 0 g Gemini: 0g DeepSeek: 0g Claude: 1g | 0.9 g | 100 g spinach | ChatGPT: 3.5 g Gemini: 3.6 g DeepSeek: 3g Claude: 2g | 3.5 g |  |  |  | ChatGPT: 32.5 g Gemini: 32.4 g DeepSeek: 33g Claude: 48g | 33.44 g |
| 65 | Dinner | 150 g omelette with 30 g wholemeal bread and 150 g lettuce | 150 g omelette | ChatGPT: 1.5 g Gemini: 0 g DeepSeek: 0g Claude: 2g | 0 | 30 g wholemeal bread | ChatGPT: 13 g Gemini: 12g DeepSeek: 15g Claude: 18g | 12.3 g | 150 g lettuce | ChatGPT: 2.5 g Gemini: 4.35. DeepSeek: 5g Claude: 3g | 4.5 g |  |  |  | ChatGPT: 17 g Gemini: 16.36 g DeepSeek: 20g Claude: 23g | 16.8 g |
| 66 | Dinner | 100 g plaice with 150 g potatoes and 80 g peaches | 100 g plaice | ChatGPT: 0 g Gemini: 0g DeepSeek: 0g Claude: 0g | 0.8 g | 150 g potatoes | ChatGPT: 25.5 g Gemini: 26.25g DeepSeek: 27g Claude: 26g | 25.5 g | 80g peaches | ChatGPT: 7.2 g Gemini: 6.4. DeepSeek: 8g Claude: 12g | 4.88 g |  |  |  | ChatGPT: 32.7 g Gemini: 32.65g DeepSeek: 35g Claude: 38g | 31.18 g |
| 67 | Dinner | 150 g minestrone (12 vegetables. Orogel) with 30 g fusilli (Barilla) and 10 g apricots | 150 g minestrone (12 vegetables. Orogel) | ChatGPT: 7.5 g Gemini: 9g DeepSeek: 12g Claude: 15 g | 9.8 g | 30 g fusilli (Barilla) | ChatGPT: 21.5 g Gemini: 21.3 g DeepSeek: 22g Claude: 20g | 21.36 g | 10g apricocs | ChatGPT: 0.9 g Gemini: 1.2g DeepSeek: 1g Claude: 2g | 0.7 g |  |  |  | ChatGPT: 29.9g Gemini: 31.5g DeepSeek: 35g Claude: 37g | 31.86 g |
| 68 | Dinner | 120 g boiled eggs with 150 g lettuce and 100 g figs | 120 g boiled eggs | ChatGPT: 0g Gemini: 0g DeepSeek: 0g Claude: 1g | 0 | 150 g lettuce | ChatGPT: 2.5 g Gemini: 4.35 g DeepSeek: 0g Claude: 2g | 4.5 g | 100g figs | ChatGPT: 16 g Gemini: 16 g DeepSeek: 19g Claude:16g | 14.2 g |  |  |  | ChatGPT: 18.5 g Gemini: 20.35g DeepSeek: 19g Claude:19g | 18.7 g |
| 69 | Dinner | 50 g stracchino cheese (Nonno Nanni) with 40 g rye bread and 120 g apple | 50 g stracchino cheese (Nonno Nanni) | ChatGPT: 1 g Gemini: 1.3g DeepSeek: 0g Claude: 1g | 1.3 g | 40 g rye bread | ChatGPT: 17 g Gemini: 18g DeepSeek: 18g Claude: 20g | 18.16 g | 120g apple | ChatGPT: 14.4 g Gemini: 12g DeepSeek: 16g Claude: 17g | 14.4 g |  |  |  | ChatGPT: 32.4 g Gemini: 31.3. DeepSeek: 34g Claude: 38g | 33.86 g |
| 70 | Dinner | 80 g cooked rice salad with tuna and olives + 80 g banana | 80 g cooked rice | ChatGPT: 22.4 g Gemini: 22.4 g DeepSeek: 20g Claude: 20g | 22.4 g | 80g banana | ChatGPT: 18.4 g Gemini: 17.6g DeepSeek: 18g Claude: 19g | 13.92 g |  |  |  |  |  |  | ChatGPT: 40.8 g Gemini: 40g DeepSeek: 38g Claude: 39g | 36.32 g |
| 71 | Dinner | 200 g vegetable velouté (Findus creamy peas & zucchini) with 20 g croutons (San Carlo) and 60 g pear | 200 g vegetable velouté (Findus creamy peas & zucchini) | ChatGPT: 12 g Gemini: 12 g DeepSeek: 20g Claude: 8g | 12.6 g | croutons (San Carlo) | ChatGPT: 14g Gemini: 14 g DeepSeek: 12g Claude: 12g | 10.4 g | 60g pear | ChatGPT:9 g Gemini: 9g DeepSeek: 9g Claude: 8g | 6.12 g |  |  |  | ChatGPT: 35 g Gemini: 35g DeepSeek: 41g Claude: 28g | 29.12 g |
| 72 | Dinner | 80g di omelette con 150 g carrots e 40 g of white bread | 80g omelette | ChatGPT: 2 g Gemini: 0g DeepSeek: 0g Claude: 2g | 0 | 150 g carrots | ChatGPT: 10.5 g Gemini: 10.5 g DeepSeek: 12g Claude: 12g | 11.4 g | 40 g white bread | ChatGPT: 20 g Gemini: 23.8 g DeepSeek: 20g Claude: 30g | 23.84 g |  |  |  | ChatGPT: 32.5 g Gemini: 34.3 g DeepSeek: 32g Claude: 44g | 35.24 g |
| 73 | Dinner | 100 g chicken thigh with 150 g grilled eggplant and 40 g rye bread | 100 g chicken thigh | ChatGPT: 0 g Gemini: 0g DeepSeek: 0g Claude:0g | 0 | 150 g grilled eggplant | ChatGPT:9 g Gemini: 8.7 g DeepSeek: 9g Claude: 6g | 8.1 g | 40 g rye bread | ChatGPT: 17 g Gemini: 18 g DeepSeek: 18g Claude: 20g | 18.16 g |  |  |  | ChatGPT: 26 g Gemini: 26.7 g DeepSeek: 27g Claude: 26 g | 26.26 g |
| 74 | Dinner | 100 g ricotta (Fattorie Osella) with 150 g spinach and 40 g white bread | 100 g ricotta (Fattorie Osella) | ChatGPT: 3g Gemini: 3 g DeepSeek: 0g Claude: 3g | 3.3 g | 150 g spinach | ChatGPT: 5.3 g Gemini: 5.4 g DeepSeek: 4g Claude: 3g | 5.25 g | 40 g white bread | ChatGPT: 22 g Gemini: 23.8g DeepSeek: 20g Claude: 30g | 23.84 g |  |  |  | ChatGPT: 30.3 g Gemini: 32.2 g DeepSeek: 24g Claude: 36g | 32.39 g |
| 75 | Dinner | 100 g wurstel (Aia) with 150 g potatoes and 70 g melon | 100 g wurstel (Aia) | ChatGPT: 1 g Gemini: 2.5g DeepSeek: 0g Claude: 2g | 0 | 150 g potatoes | ChatGPT: 25.5 g Gemini: 26.25. DeepSeek: 27g Claude: 26g | 25.5 g | 70g melon | ChatGPT: 6 g Gemini: 5.6g DeepSeek: 6g Claude: 5g | 5.18 g |  |  |  | ChatGPT: 32.5 g Gemini: 34.35g DeepSeek: 33g Claude: 33g | 30.68 g |
| 76 | Dinner | 80 g zucchini au gratin with 80 g white bread | 80 g zucchini au gratin | ChatGPT:5 g Gemini: 2.48g DeepSeek: 3g Claude: 6g | 4.16 g | 80 g white bread | ChatGPT: 44 g Gemini: 47.6 g DeepSeek: 40g Claude: 60g | 47.68 g |  |  |  |  |  |  | ChatGPT: 49 g Gemini: 50.08g DeepSeek: 43 g Claude: 66g | 51.84 g |
| 77 | Dinner | 100 g chicken breast with 150 g cabbage and 120 g apple | 100 g chicken breast | ChatGPT: 0 g Gemini: 0g DeepSeek: 0g Claude:0g | 0 | 150 g cabbage | ChatGPT: 7.5g Gemini: 7.5g DeepSeek: 9g Claude: 7g | 5.3 g | 120g apple | ChatGPT: 14.4 g Gemini: 12g DeepSeek: 16g Claude:17g | 14.4 g |  |  |  | ChatGPT: 21.9 g Gemini: 19.5g DeepSeek: 25g Claude: 24g | 19.7 g |
| 78 | Dinner | 50 g fresh pecorino with 40 g white bread and 150 g lettuce | 50 g fresh pecorino | ChatGPT: 0.4g Gemini:0g DeepSeek: 0g Claude: 0g | 0.1 g | 40 g white bread | ChatGPT: 22 g Gemini: 23.8g DeepSeek: 20g Claude:30g | 23.84 g | 150 g di lettuce | ChatGPT: 2.5 g Gemini: 4.35g DeepSeek: 3g Claude: 3g | 4.5 g |  |  |  | ChatGPT: 24.9 g Gemini: 28.15. DeepSeek: 23g Claude: 33g | 28.44 g |
| 79 | Dinner | 80 g tuna (Nostromo) with 150 g green beans and 40 g rye bread | 80 g tuna (Nostromo) | ChatGPT: 0 g Gemini: 0g DeepSeek: 0g Claude:0g | 0 | 150 g green beans | ChatGPT: 6.5 g Gemini: 10.5. DeepSeek: 9g Claude: 7g | 3.6 g | 40 g rye bread | ChatGPT: 17 g Gemini: 18g DeepSeek: 18g Claude: 20g | 18.16 g |  |  |  | ChatGPT: 23.5 g Gemini: 28.5g DeepSeek: 27g Claude: 27g | 21.76 g |
| 80 | Dinner | 200 g vegetable purée (Orogel Verdurì selection) with 20 g white bread | 200 g vegetable purée (Orogel Verdurì selection) | ChatGPT: 9 g Gemini: 10g DeepSeek: 14g Claude: 15g | 8 | 20 g white bread | ChatGPT: 10 g Gemini: 11.9. DeepSeek: 10g Claude:12g | 11.92 g |  |  |  |  |  |  | ChatGPT: 19 g Gemini: 21.9g DeepSeek: 24g Claude: 27g | 19.92 g |
| 81 | Dinner | 150 g white pizza | 150 g white pizza | ChatGPT: 45 g Gemini: 42g DeepSeek: 60g Claude: 45g | 86.8 g |  |  |  |  |  |  |  |  |  | ChatGPT: 45 g Gemini: 42g DeepSeek: 60g Claude: 45g | 86.9 g |
| 82 | Dinner | 100 g risotto (parboiled raw) with saffron and 100 g peas (Primavera. Findus) | 100 g risotto (parboiled) with saffron | ChatGPT: 78 g Gemini: 81.3 g DeepSeek: 78g Claude: 73 g | 81.3 g | 100 g peas (Primavera. Findus) | ChatGPT: 10 g Gemini: 7.5 g DeepSeek: 11 g Claude: 6 g | 7.5 g |  |  |  |  |  |  | ChatGPT: 88 g Gemini: 88.8 g DeepSeek: 89 g Claude: 79 g | 88.8 g |
| 83 | Dinner | 150 g beef meatballs with 100 g mashed potatoes | 150 g beef meatballs | ChatGPT: 9 g Gemini: 11.25 g DeepSeek: 0g Claude: 0g | 7.58 g | 100 g mashed potatoes ( ) Dimmidisì | ChatGPT: 15 g Gemini: 15 g DeepSeek: 20g Claude: 18g | 13 |  |  |  |  |  |  | ChatGPT: 24 g Gemini: 26.25g DeepSeek: 20g Claude: 18g | 20.58 g |
| 84 | Dinner | 150 g chicken salad with 50 g avocado and 40 g white bread | 150 g chicken salad | ChatGPT: 2 g Gemini: 0 g DeepSeek: 0g Claude: 0g | 0.5 g | 50 g avocado | ChatGPT:2 g Gemini: 4.25g DeepSeek: 2g Claude: 2g | 0.9 g | 40 g white bread | ChatGPT: 20 g Gemini: 23.8g DeepSeek: 20g Claude: 30g | 23.84 g |  |  |  | ChatGPT: 24g Gemini: 28.5 g DeepSeek: 22g Claude: 32g | 25.24 g |
| 85 | Dinner | 100 g omelette and 40 g parmesan | 100 g omelette | ChatGPT: 1 g Gemini: 0 g DeepSeek: 0g Claude: 2g | 0 | 40 g parmesan | ChatGPT: 0 g Gemini: 0g DeepSeek: 0g Claude: 0g | 0 |  |  |  |  |  |  | ChatGPT: 1 g Gemini: 0 g DeepSeek: 0g Claude: 2g | 0 |
| 86 | Dinner | 200 g chicken broth with 35 g pastina | 200 g chicken broth | ChatGPT: 0 g Gemini: 0g DeepSeek: 0g Claude: 0g | 0 | 35 grammi pastina | ChatGPT: 26 g Gemini: 27.65 g DeepSeek: 26 g Claude: 25 g | 25.27 g |  |  |  |  |  |  | ChatGPT: 26 g Gemini: 27.65 g DeepSeek: 26 g Claude: 25 g | 25.27 g |
| 87 | Dinner | 80 g short pasta (Molisana) with 50 g tomato sauce (Mutti) | 80 g short pasta (Molisana) | ChatGPT: 56 g Gemini: 56 g DeepSeek: 60g Claude: 50g | 56 | 50 g tomato sauce (Mutti) | ChatGPT: 2 g Gemini: 1.75 g DeepSeek: . Claude: 3g | 2.55 g |  |  |  |  |  |  | ChatGPT: 58 g Gemini: 57.75 g DeepSeek: 63g Claude: 53g | 58.55 g |
| 88 | Dinner | 100 g baked chicken with 100 g Swiss chard | 100 g baked chicken | ChatGPT: 0 g Gemini: 0g DeepSeek: 0g Claude: 0g | 0 | 100 g Swiss chard | ChatGPT: 2 g Gemini: 3g DeepSeek: 3g Claude: 3g | 2.1 g |  |  |  |  |  |  | ChatGPT: 2 g Gemini: 3 g DeepSeek: 3g Claude: 3g | 2.1 g |
| 89 | Dinner | 100 g cornmeal polenta with 50 g pecorino | 100 g of polenta (raw) | ChatGPT: 73 g Gemini: 77 g DeepSeek: 75 g Claude: 73g | 72.7g | 50 g pecorino | ChatGPT: 0 g Gemini: 0g DeepSeek: 0g Claude: 0.5 g | 0.1 g |  |  |  |  |  |  | ChatGPT: 73 g Gemini: 77 g DeepSeek: 75 g Claude: 73g | 72.8 g |
| 90 | Dinner | 200 g fish soup (fish. tomato. vegetables) | 200 g fish soup | ChatGPT: 5.4 g Gemini: 15 g DeepSeek: 12g Claude: 10g | 5.3 g |  |  |  |  |  |  |  |  |  | ChatGPT: 5.4 g Gemini: 15 g DeepSeek: 12g Claude: 10g | 5.3 g |
| 91 | Dinner | 200 g sliced beef with 150 g iceberg lettuce | 200 g sliced beef | ChatGPT: 0 g Gemini: 0g DeepSeek: 0g Claude: 0g | 0 | 150 g iceberg lettuce | ChatGPT: 2.5 g Gemini: 4.35 g DeepSeek: 3 g Claude: 2.4 g | 4.5 g |  |  |  |  |  |  | ChatGPT: 2.5 g Gemini: 4.35 g DeepSeek: 3 g Claude: 2.4 g | 4.5 g |
| 92 | Dinner | 80 g couscous with 20 g peppers. 20 g zucchini. 20 g onions | 80 g couscous | ChatGPT: 59 g Gemini: 63.2 g DeepSeek: 62 g Claude: 58 g | 63.2 g | 60 g of peppers. zucchini. and onions (20 g of each vegetable) | ChatGPT: 3.7 g Gemini: 3.62g DeepSeek: 3.5 g Claude: 1 g | 2.78 g |  |  |  |  |  |  | ChatGPT: 62.7 g Gemini: 66.8 g DeepSeek: 63 g Claude: 59 g | 65.98 g |
| 93 | Dinner | 100 g chicken curry with 50 g spinach | 100 g chicken curry | ChatGPT: 0 g Gemini: 5g DeepSeek: 0g Claude:0g | 0 | 50 g spinach | ChatGPT: 0.55 g Gemini: 1.8 g DeepSeek: 2g Claude: 1g | 1.82 g |  |  |  |  |  |  | ChatGPT: 0.6 g Gemini: 6.8 g DeepSeek: 5g Claude: 1g | 1.82 g |
| 94 | Snacks | 150 g banana | 150 g banana | ChatGPT: 33 g Gemini: 33g DeepSeek: 34g Claude: 27g | 26 |  |  |  |  |  |  |  |  |  | ChatGPT: 34.5 g Gemini: 33g DeepSeek: 34g Claude: 27g | 26 |
| 95 | Snacks | 200 ml pear juice (Zueg) | 200 ml pear juice (Zueg) | ChatGPT: 20 g Gemini: 16g DeepSeek: 18g Claude: 24g | 26 |  |  |  |  |  |  |  |  |  | ChatGPT: 15.6 g Gemini: 16g DeepSeek: 18g Claude: 24g | 26 |
| 96 | Snacks | 125 g low-fat cherry yogurt (Yomo) | 125 g low-fat cherry yogurt YOMO | ChatGPT:15 g Gemini: 17 g DeepSeek: 16.3g Claude: 18.5g | 15 |  |  |  |  |  |  |  |  |  | ChatGPT:15 g Gemini: 17 g DeepSeek: 16.3g Claude: 18.5g | 15 |
| 97 | Snacks | 15 g wholemeal rusks (Mulino Bianco) | 15 g wholemeal rusks (Mulino Bianco) | ChatGPT: 10 g Gemini: 10.13g DeepSeek: 10g Claude: 8.5g | 9 |  |  |  |  |  |  |  |  |  | ChatGPT: 10g Gemini: 10.13g DeepSeek: 10g Claude: 8.5g | 9 |
| 98 | Snacks | 30 g wholemeal bread with 10 g honey | 30 g wholemeal bread | ChatGPT: 12 g Gemini: 12g DeepSeek: 14g Claude: 12.5g | 14.5 g | 10 g di miele | ChatGPT: 8g Gemini: 8g DeepSeek: 8g Claude: 8g | 8 |  |  |  |  |  |  | ChatGPT: 20g Gemini: 20g DeepSeek: 22g Claude: 20.5g | 22.5 g |
| 99 | Snacks | 20 g cereal bar (chocolate. Special K) | 20 g cereal bar (chocolate. Special K) | ChatGPT: 14.6g Gemini: 13g DeepSeek: 14g Claude: 12g | 12 |  |  |  |  |  |  |  |  |  | ChatGPT: 14.6 g Gemini: 13g DeepSeek: 14g Claude: 12g | 12 |
| 100 | Snacks | 40 g white bread with 10 g quince jam | 40 g white bread | ChatGPT: 20 g Gemini: 23.8g DeepSeek: 20g Claude: 17g | 23.84 g | 10 g quince jam | ChatGPT: 6.5 g Gemini: 6.5g DeepSeek: 7g Claude: 5g | 7.8 g |  |  |  |  |  |  | ChatGPT: 26.5 g Gemini: 30.3g DeepSeek: 27g Claude: 22g | 31.64 g |
| 101 | Snacks | 125 g plain yogurt + 10 g sugar | 125 g plain yogurt | ChatGPT: 5.6 g Gemini: 6.25g DeepSeek: 5g Claude: 4g | 5.5 g | 10 g sugar | ChatGPT: 10 g Gemini: 10g DeepSeek: 10g Claude: 8g | 10 |  |  |  |  |  |  | ChatGPT: 15.6g Gemini: 16.25 g DeepSeek: 15g Claude: 12g | 15.5 g |
| 102 | Snacks | 30 g crackers (Mulino Bianco) | 30 g crackers (Mulino Bianco) | ChatGPT: 20 g Gemini: 18g DeepSeek: 20g Claude: 20g | 20.4 g |  |  |  |  |  |  |  |  |  | ChatGPT: 20 g Gemini: 18g DeepSeek: 20g Claude: 20g | 20.4 g |
| 103 | Snacks | 100 g banana and 15 g biscuits (Oro Saiwa) | 100 g of banana | ChatGPT: 22g Gemini: 22g DeepSeek: 23g Claude: 19g | 17.4 g | 15 g biscuits (Oro Saiwa) | ChatGPT: 11.1 g Gemini: 11.25. DeepSeek: 10g Claude: 10g | 11.1 g |  |  |  |  |  |  | ChatGPT: 33.1 g Gemini: 33.25g DeepSeek: 33g Claude: 29g | 28.5 g |
| 104 | Snacks | 150 g apple and 7 g rusks (Mulino Bianco) | 150 g apple | ChatGPT: 18 g Gemini: 15g DeepSeek: 17g Claude: 14g | 16.7 g | 7 g rusks (Mulino Bianco) | ChatGPT:4.7 g Gemini: 5.25g DeepSeek: 5g Claude: 6g | 4.6 g |  |  |  |  |  |  | ChatGPT: 22.7g Gemini: 20.25 g DeepSeek: 22g Claude: 20g | 21.3 g |
| 105 | Snacks | 200 ml pear juice (Santal) and 14 g rusks (Mulino Bianco) | 200 ml pear juice (Santal) | ChatGPT: 20g Gemini: 20g DeepSeek: 22g Claude: 20g | 28 | 14 g rusks (Mulino Bianco) | ChatGPT: 9.4 g Gemini: 10.5g DeepSeek: 10g Claude: 10g | 9.2 g |  |  |  |  |  |  | ChatGPT: 29.4g Gemini: 30.5 g DeepSeek: 32g Claude: 30g | 37.2 g |
| 106 | Snacks | 125 g strawberry yogurt and 100 g figs | 125 g strawberry yogurt | ChatGPT: 15 g Gemini: 21.88g DeepSeek: 18g Claude: 18g | 13 | 100 g figs | ChatGPT: 16 g Gemini: 16 g DeepSeek: 19g Claude: 12g | 14.2 g |  |  |  |  |  |  | ChatGPT: 31 g Gemini: 37.88 g DeepSeek: 37g Claude: 30g | 27.2 g |
| 107 | Snacks | 40 g white bread with 5 g olive oil | 40 g white bread | ChatGPT: 20 g Gemini: 23.8g DeepSeek: 20g Claude: 24g | 23.84 g | 5 g olive oil | ChatGPT: 0 g Gemini: 0g DeepSeek: 0g Claude: 0g | 0 |  |  |  |  |  |  | ChatGPT: 20 g Gemini: 23.8g DeepSeek: 20g Claude: 24g | 23.84 g |
| 108 | Snacks | 50 g tart | 50 g tart | ChatGPT: 24 g Gemini: 22.5g DeepSeek: 30g Claude: 30g | 32.75 g |  |  |  |  |  |  |  |  |  | ChatGPT: 24 g Gemini: 22.5 g DeepSeek: 30g Claude: 30g | 32.75 g |
| 109 | Snacks | 150 ml semi-skimmed milk (Latteria) and 20 g Pavesini biscuit | 150 ml semi-skimmed milk (Latteria) | ChatGPT: 7.5 g Gemini: 7.5g DeepSeek: 7g Claude: 7g | 7.35 g | 20 g Pavesini biscuit | ChatGPT: 15.6g Gemini: 16 g DeepSeek: 14g Claude: 16g | 16.6 g |  |  |  |  |  |  | ChatGPT: 23.1 g Gemini: 23.5 g DeepSeek: 21g Claude: 23g | 23.95 g |
| 110 | Snacks | 10 g honey and 125 g whole yogurt | 10 g honey | ChatGPT: 8 g Gemini: 8g DeepSeek: 8g Claude:8g | 8 | 125 g whole yogurt | ChatGPT: 5.9 g Gemini: 5.63g DeepSeek: 5g Claude:4g | 5.5 g |  |  |  |  |  |  | ChatGPT: 13.9 g Gemini: 13.63 g DeepSeek: 13g Claude: 12g | 13.5 g |
| 111 | Snacks | 150 g apple and 15 g biscuits (Macine. Mulino Bianco) | 150 g apple | ChatGPT: 18 g Gemini: 15g DeepSeek: 17g Claude: 14g | 16.7 g | 15 g biscuits (Macine. Mulino Bianco) | ChatGPT: 9.7 g Gemini: 10.5 g DeepSeek: 10g Claude: 10g | 10.2 g |  |  |  |  |  |  | ChatGPT: 27.7 g Gemini: 25.5 g DeepSeek: 27g Claude: 24g | 26.9 g |
| 112 | Snacks | 40 g wholemeal bread with 50 g cooked ham + 30 g cheese | 40 g wholemeal bread | ChatGPT: 16 g Gemini: 16g DeepSeek: 18g Claude: 16.6g | 16.4 g | 50 g cooked ham | ChatGPT: 0 g Gemini: 0g DeepSeek: 0g Claude: 0g | 0 | 30 g cheese | ChatGPT: 0 g Gemini: 0g DeepSeek: 0g Claude: 0g | 0 |  |  |  | ChatGPT: 16 g Gemini: 16 g DeepSeek: 18g Claude: 16.6g | 16.4 g |
| 113 | Snacks | 125 g Greek yogurt with 20 g dried fruit + 10 g honey | 125 g Greek yogurt | ChatGPT: 4.5 g Gemini: 4.38g DeepSeek: 5g Claude:4g | 2.5 g | 20 g dried fruit | ChatGPT: 1.2g Gemini: 2.5g DeepSeek: 4g Claude: 4g | 2 | 10 g honey | ChatGPT: 8.2. Gemini: 8g DeepSeek: 8g Claude:8g | 8 |  |  |  | ChatGPT: 13.9 g Gemini: 14.88 g DeepSeek: 17g Claude: 16g | 12.5 g |
| 114 | Snacks | 150 g banana + 15 g peanut butter | 150g banana | ChatGPT: 33g Gemini: 33 g DeepSeek: 34g Claude: 27g | 26 | 15 g peanut butter marca fiorentini | ChatGPT: 2.1 g Gemini: 2.63g DeepSeek: 3g Claude: 3g | 2.1 g |  |  |  |  |  |  | ChatGPT: 35.1 g Gemini: 35.63 g DeepSeek: 37g Claude: 30g | 28.1 g |
| 115 | Snacks | 150 ml semi-skimmed milk + 30 g wholemeal biscuits (Misura) | 150 ml semi-skimmed milk | ChatGPT: 7.5 g Gemini: 7.5g DeepSeek: 7g Claude:7g | 7.5 g | 30 g wholemeal biscuits (Misura) | ChatGPT: 19.5 g Gemini: 18.75g DeepSeek: 18g Claude: 20g | 20.1 g |  |  |  |  |  |  | ChatGPT: 27 g Gemini: 26.25 g DeepSeek: 25g Claude: 27g | 27.6 g |
| 116 | Snacks | 30 g wholemeal bread with 50 g avocado + 50 g boiled egg | 30 g wholemeal bread | ChatGPT: 12.9 g Gemini: 12g DeepSeek: 14g Claude: 12.5g | 12.3 g | 50 g avocado | ChatGPT: 1 g Gemini: 4.25g DeepSeek: 2g Claude: 5g | 0.9 g | 50 g boiled egg | ChatGPT: 0 g Gemini: 0g DeepSeek: 0g Claude: 0g | 0 |  |  |  | ChatGPT: 13.9 g Gemini: 16.25 g DeepSeek: 16g Claude: 17.5g | 13.2 g |
| 117 | Snacks | 70 g kiwi with 20 g walnuts | 70 g kiwi | ChatGPT: 10.22 g Gemini: 10.5g DeepSeek: 8g Claude:6g | 9.4 g | 20 g walnuts | ChatGPT: 0.66 g Gemini: 2.8g DeepSeek: 2g Claude:2g | 1 |  |  |  |  |  |  | ChatGPT: 10.9 g Gemini: 13.3 g DeepSeek: 10g Claude: 8g | 10.4 g |
| 118 | Snacks | 100 ml milk (Zymil) with 10 g cocoa powder + 14 g rusks | 100 ml milk (Zymil) | ChatGPT: 5 g Gemini: 5g DeepSeek: 5g Claude:5g | 4.9 g | 10 g cocoa powder | ChatGPT: 0.45 g Gemini: 1.25g DeepSeek: 2g Claude: 2g | 1.2 g | 14 g rusks | ChatGPT: 11 g Gemini: 10.5g DeepSeek: 10g Claude:12g | 10.5 g |  |  |  | ChatGPT: 16.5 g Gemini: 16.75 g DeepSeek: 17g Claude:19 g | 16.6 g |
| 119 | Snacks | 30 g protein bar (MyProtein-High Protein) + 200 ml pear juice (Santal) | 30 g protein bar My protein Barretta High Pro | ChatGPT: 9 g Gemini: 9g DeepSeek: 9g Claude: 9 g | 8.7 g | 200 ml pear juice (Santal) quale? | ChatGPT: 28 g Gemini: 22 g DeepSeek: 22g Claude: 22g | 28 |  |  |  |  |  |  | ChatGPT: 37 g Gemini: 37g DeepSeek: 37. Claude: 37 g | 36.7 g |
| 120 | Snacks | 50 ml cappuccino + 50 g plain croissant (bakery) | 50 ml cappuccino | ChatGPT: 1.7 g Gemini: 2.5g DeepSeek: 3g Claude:3g | 2.5 g | 50 g plain croissant (bakery) | ChatGPT: 25 g Gemini: 25g DeepSeek: 25g Claude:27g | 25 |  |  |  |  |  |  | ChatGPT: 26.7 g Gemini: 27.5g DeepSeek: 28g Claude: 30g | 27.5 g |
| 121 | Snacks | 40 g wholemeal bread with 50 g cured ham + 30 g aged cheese | 40 g wholemeal bread | ChatGPT: 17.2 g Gemini: 16 g DeepSeek: 18g Claude: 16.6g | 16.4 g | 50 g cured ham | ChatGPT: 0 g Gemini: 0g DeepSeek: 0g Claude: 0g | 0 | 30 g aged cheese | ChatGPT: 0 g Gemini: 0g DeepSeek: 0g Claude: 0g | 0 |  |  |  | ChatGPT: 17.2g Gemini: 16g DeepSeek: 18g Claude: 16.6g | 16.4 g |
| 122 | Snacks | Fruit smoothie (150 ml milk + 100 g banana + 10 g honey) | 150 ml of milk | ChatGPT: 7.5 g Gemini: 7.5 g DeepSeek: 7g Claude: 7g | 7.5 g | 100 g banana | ChatGPT: 22 g Gemini: 22g DeepSeek: 23 g Claude: 19g | 17.4 g | 10 g honey | ChatGPT: 8.2. Gemini: 8g DeepSeek: 8g Claude: 8g | 8 |  |  |  | ChatGPT: 37.5 g Gemini: 37.5g DeepSeek: 38g Claude: 34g | 32.9 g |
| 123 | Snacks | 50 g lemon cake (Mulino Bianco) + 200 ml pear juice (Santal) | 50 g lemon cake (Mulino Bianco) | ChatGPT: 30g Gemini: 25g DeepSeek: 32g Claude: 28g | 24.8 g | 200 ml pear juice (Santal) | ChatGPT: 25 g Gemini: 22 g DeepSeek: 22g Claude: 22g | 28 |  |  |  |  |  |  | ChatGPT: 55g Gemini: 43.8 g DeepSeek: 54g Claude: 50g | 52.8 g |
| 124 | Snacks | 30 g white bread with 50 g smoked salmon + 20 g cream cheese | 30 g white bread | ChatGPT: 15g Gemini: 17.85g DeepSeek: 15g Claude: 21g | 17.88 g | 50 g smoked salmon | ChatGPT: 0 g Gemini: 0g DeepSeek: 0g Claude: 0g | 0 | 20 g cream cheese | ChatGPT: 0.6g Gemini: 0.9g DeepSeek: 0g Claude: 1.2g | 0 |  |  |  | ChatGPT: 15.6 g Gemini: 18.75g DeepSeek: 15g Claude: 22.2g | 17.88 g |

Commercial brand names mentioned in this table include Parmalat™. Müller™. Yoga™. Mulino Bianco™. Misura™. Nesquik™. Alpro™. Yomo™. Zymil™. Ferrero™. Nutella™. Pavesi™. Pavesini™. Gocciola™. Bauli™. McDonald’s™. Coca-Cola™. Certosa™. Philadelphia™. Vallelata™. Barilla™. Gallo™. COOP™. Cucina Esselunga™. DimmidiSì™. Galbani™. De Cecco™. Rana™. Orogel™. Verdurì™. Findus™. Primavera™. San Carlo™. Nonno Nanni™. Fattorie Osella™. AIA™. Nostromo™. Molisana™. Mutti™. Zuegg™. Special K™. Oro Saiwa™. Santal™. Latteria™. Macine™. and MyProtein™. All trademarks belong to their respective owners and are reported for identification purposes only.
