# Supplementary material for: Accuracy of ChatGPT, Gemini, Claude and DeepSeek in Carbohydrate Counting
Source: Diabetes Obes Metab. 2026 Apr 13;28(7):5627–36. doi: 10.1111/dom.70747 (PMC13243987; doi:10.1111/dom.70747)
Supplement: Supplementary file 6 — Table S3: Model performance according to clinically interpretable absolute‐error thresholds and carbohydrate‐load strata (< 20, 20–50, > 50 g). [file DOM-28-5627-s008.docx]

| **CHO-load stratum** | **Model** | **n** | **Rate within ±5 g** | **Rate within ±10 g** | **Rate within ±15 g** | **Rate >10 g** | **MAE, g** | **Bias, g** |
| --- | --- | --- | --- | --- | --- | --- | --- | --- |
| Overall | ChatGPT | 124 | 82.3 | 98.4 | 100.0 | 1.6 | 2.71 | -0.30 |
| Overall | Gemini | 124 | 78.2 | 91.1 | 93.5 | 8.9 | 4.79 | -1.81 |
| Overall | DeepSeek | 124 | 74.2 | 89.5 | 91.9 | 10.5 | 5.46 | -1.12 |
| Overall | Claude | 124 | 73.4 | 88.7 | 91.1 | 11.3 | 6.03 | -2.55 |
| <20 g | ChatGPT | 30 | 96.7 | 100.0 | 100.0 | 0.0 | 1.54 | 0.87 |
| <20 g | Gemini | 30 | 90.0 | 100.0 | 100.0 | 0.0 | 1.58 | 1.11 |
| <20 g | DeepSeek | 30 | 86.7 | 96.7 | 100.0 | 3.3 | 2.50 | 1.43 |
| <20 g | Claude | 30 | 86.7 | 100.0 | 100.0 | 0.0 | 2.59 | 0.66 |
| 20-50 g | ChatGPT | 70 | 82.9 | 98.6 | 100.0 | 1.4 | 2.77 | -0.34 |
| 20-50 g | Gemini | 70 | 78.6 | 94.3 | 98.6 | 5.7 | 3.20 | 0.69 |
| 20-50 g | DeepSeek | 70 | 77.1 | 92.9 | 95.7 | 7.1 | 4.00 | 0.92 |
| 20-50 g | Claude | 70 | 81.4 | 95.7 | 98.6 | 4.3 | 3.24 | 0.15 |
| >50 g | ChatGPT | 24 | 62.5 | 95.8 | 100.0 | 4.2 | 4.00 | -1.67 |
| >50 g | Gemini | 24 | 62.5 | 70.8 | 70.8 | 29.2 | 13.45 | -12.76 |
| >50 g | DeepSeek | 24 | 50.0 | 70.8 | 70.8 | 29.2 | 13.41 | -10.28 |
| >50 g | Claude | 24 | 33.3 | 54.2 | 58.3 | 45.8 | 18.50 | -14.45 |

**Supplementary Table 3.**

Model performance according to clinically interpretable absolute-error thresholds and carbohydrate-load strata (<20 g, 20-50 g, >50 g)

Absolute-error thresholds are expressed in grams relative to the clinicians' reference value. "Rate >10 g" indicates the proportion of meals with an absolute error >10 g. Carbohydrate-load strata were defined according to the clinicians' reference carbohydrate content: <20 g, 20-50 g, and >50 g. MAE = mean absolute error. Bias = mean signed difference (model - reference).

Statistical comparison of model performance according to clinically interpretable absolute-error thresholds and carbohydrate-load strata.

**Panel A. Global comparisons across the four models**

| **Analysis** | **Stratum** | **Test** | **Statistic** | **p value** |
| --- | --- | --- | --- | --- |
| Rate of meals within ±5 g | Overall | Cochran's Q | 5.37 | 0.146 |
| Rate of meals within ±10 g | Overall | Cochran's Q | 15.88 | 0.0012 |
| Rate of meals within ±15 g | Overall | Cochran's Q | 21.88 | <0.001 |
| Absolute error | Overall | Friedman test | 11.53 | 0.0092 |
| Rate of meals within ±10 g | <20 g | Cochran's Q | 3.00 | 0.392 |
| Absolute error | <20 g | Friedman test | 11.60 | 0.0089 |
| Rate of meals within ±10 g | 20-50 g | Cochran's Q | 3.62 | 0.305 |
| Absolute error | 20-50 g | Friedman test | 2.34 | 0.505 |
| Rate of meals within ±10 g | >50 g | Cochran's Q | 17.00 | 0.00071 |
| Absolute error | >50 g | Friedman test | 10.47 | 0.0149 |

**Panel B. Pairwise comparisons for the proportion of meals within ±10 g in the overall dataset**

| **Comparison** | **Test** | **Unadjusted p value** | **Bonferroni-adjusted p value** |
| --- | --- | --- | --- |
| ChatGPT vs Gemini | McNemar exact | 0.0225 | 0.135 |
| ChatGPT vs DeepSeek | McNemar exact | 0.0074 | 0.044 |
| ChatGPT vs Claude | McNemar exact | 0.0042 | 0.025 |
| Gemini vs DeepSeek | McNemar exact | 0.6875 | 1.000 |
| Gemini vs Claude | McNemar exact | 0.5078 | 1.000 |
| DeepSeek vs Claude | McNemar exact | 1.000 | 1.000 |

**Panel C. Pairwise comparisons for the proportion of meals within ±10 g in the >50 g stratum**

| **Comparison** | **Test** | **Unadjusted p value** | **Bonferroni-adjusted p value** |
| --- | --- | --- | --- |
| ChatGPT vs Gemini | McNemar exact | 0.0703 | 0.422 |
| ChatGPT vs DeepSeek | McNemar exact | 0.0703 | 0.422 |
| ChatGPT vs Claude | McNemar exact | 0.0063 | 0.0381 |
| Gemini vs DeepSeek | McNemar exact | 1.000 | 1.000 |
| Gemini vs Claude | McNemar exact | 0.125 | 0.750 |
| DeepSeek vs Claude | McNemar exact | 0.125 | 0.750 |

Cochran's Q test was used for global comparison of paired binary outcomes across the four models. McNemar exact test was used for pairwise post hoc comparisons. Friedman test was used for paired comparison of continuous absolute-error values across the four models. Pairwise post hoc analyses were restricted to the ±10 g threshold because this was considered the most clinically interpretable absolute-error cut-off. Carbohydrate-load strata were defined according to the clinicians' reference carb
