# Supplementary material for: Accuracy of ChatGPT, Gemini, Claude and DeepSeek in Carbohydrate Counting
Source: Diabetes Obes Metab. 2026 Apr 13;28(7):5627–36. doi: 10.1111/dom.70747 (PMC13243987; doi:10.1111/dom.70747)
Supplement: Supplementary file 7 — Table S4: Clinical translation of carbohydrate‐counting error into theoretical and relative insulin‐dose differences overall and in meals containing > 50 g of carbohydrates. [file DOM-28-5627-s004.docx]

**Supplementary Table 4**

Clinical translation of carbohydrate-counting error into theoretical and relative insulin-dose differences overall and in meals containing >50 g of carbohydrates.

**Panel A. Overall dataset - mean absolute theoretical insulin-dose differences**

| **Model** | **MAE, g** | **Mean absolute theoretical dose difference, U (ICR 1:8)** | **Mean absolute theoretical dose difference, U (ICR 1:10)** | **Mean absolute theoretical dose difference, U (ICR 1:15)** |
| --- | --- | --- | --- | --- |
| ChatGPT | 2.71 | 0.34 | 0.27 | 0.18 |
| Gemini | 4.79 | 0.60 | 0.48 | 0.32 |
| DeepSeek | 5.46 | 0.68 | 0.55 | 0.36 |
| Claude | 6.03 | 0.75 | 0.60 | 0.40 |

**Panel B. Meals containing >50 g of carbohydrates - mean absolute theoretical insulin-dose differences**

| **Model** | **MAE, g** | **Mean absolute theoretical dose difference, U (ICR 1:8)** | **Mean absolute theoretical dose difference, U (ICR 1:10)** | **Mean absolute theoretical dose difference, U (ICR 1:15)** |
| --- | --- | --- | --- | --- |
| ChatGPT | 4.00 | 0.50 | 0.40 | 0.27 |
| Gemini | 13.45 | 1.68 | 1.35 | 0.90 |
| DeepSeek | 13.41 | 1.68 | 1.34 | 0.89 |
| Claude | 18.50 | 2.31 | 1.85 | 1.23 |

**Panel C. Overall dataset - variability and potential excess insulin indicators in units**

| **Model** | **SD of signed carbohydrate error, g** | **IQR of absolute carbohydrate error, g** | **Meals with carbohydrate overestimation >10 g, %** | **Meals with theoretical excess insulin >0.5 U, % (ICR 1:8)** | **Meals with theoretical excess insulin >0.5 U, % (ICR 1:10)** | **Meals with theoretical excess insulin >0.5 U, % (ICR 1:15)** | **Meals with theoretical excess insulin >1.0 U, % (ICR 1:8)** | **Meals with theoretical excess insulin >1.0 U, % (ICR 1:10)** | **Meals with theoretical excess insulin >1.0 U, % (ICR 1:15)** |
| --- | --- | --- | --- | --- | --- | --- | --- | --- | --- |
| ChatGPT | 3.83 | 2.99 | 0.81 | 8.87 | 6.45 | 2.42 | 2.42 | 0.81 | 0.00 |
| Gemini | 10.23 | 3.64 | 1.61 | 12.10 | 9.68 | 3.23 | 3.23 | 1.61 | 0.81 |
| DeepSeek | 10.71 | 4.14 | 3.23 | 17.74 | 12.90 | 6.45 | 4.03 | 3.23 | 1.61 |
| Claude | 11.57 | 3.99 | 2.42 | 16.13 | 9.68 | 4.03 | 4.03 | 2.42 | 0.81 |

**Panel D. Meals containing >50 g of carbohydrates - variability and potential excess insulin indicators in units**

| **Model** | **SD of signed carbohydrate error, g** | **IQR of absolute carbohydrate error, g** | **Meals with carbohydrate overestimation >10 g, %** | **Meals with theoretical excess insulin >0.5 U, % (ICR 1:8)** | **Meals with theoretical excess insulin >0.5 U, % (ICR 1:10)** | **Meals with theoretical excess insulin >0.5 U, % (ICR 1:15)** | **Meals with theoretical excess insulin >1.0 U, % (ICR 1:8)** | **Meals with theoretical excess insulin >1.0 U, % (ICR 1:10)** | **Meals with theoretical excess insulin >1.0 U, % (ICR 1:15)** |
| --- | --- | --- | --- | --- | --- | --- | --- | --- | --- |
| ChatGPT | 5.18 | 6.63 | 4.17 | 8.33 | 8.33 | 4.17 | 4.17 | 4.17 | 0.00 |
| Gemini | 18.27 | 30.15 | 0.00 | 4.17 | 0.00 | 0.00 | 0.00 | 0.00 | 0.00 |
| DeepSeek | 18.49 | 25.43 | 0.00 | 20.83 | 12.50 | 4.17 | 0.00 | 0.00 | 0.00 |
| Claude | 21.15 | 32.39 | 8.33 | 12.50 | 12.50 | 12.50 | 12.50 | 8.33 | 4.17 |

**Panel E. Overall dataset - absolute relative insulin-dose difference (%)**

| **Model** | **Mean absolute relative insulin-dose difference, %** | **Median absolute relative insulin-dose difference, %** | **IQR of absolute relative insulin-dose difference, %** |
| --- | --- | --- | --- |
| ChatGPT | 11.12 | 7.14 | 10.63 |
| Gemini | 16.63 | 7.14 | 10.61 |
| DeepSeek | 18.60 | 8.40 | 16.36 |
| Claude | 17.44 | 9.89 | 20.12 |

**Panel F. Meals containing >50 g of carbohydrates - absolute relative insulin-dose difference (%)**

| **Model** | **Mean absolute relative insulin-dose difference, %** | **Median absolute relative insulin-dose difference, %** | **IQR of absolute relative insulin-dose difference, %** |
| --- | --- | --- | --- |
| ChatGPT | 5.93 | 4.32 | 8.33 |
| Gemini | 18.95 | 6.33 | 40.63 |
| DeepSeek | 19.39 | 8.22 | 30.97 |
| Claude | 27.34 | 12.58 | 48.28 |

**Panel G. Overall dataset - positive relative insulin-dose difference (%)**

| **Model** | **Meals with positive relative insulin-dose difference >10%, %** | **Meals with positive relative insulin-dose difference >20%, %** | **Meals with positive relative insulin-dose difference >30%, %** |
| --- | --- | --- | --- |
| ChatGPT | 17.89 | 7.32 | 4.88 |
| Gemini | 22.76 | 8.94 | 6.50 |
| DeepSeek | 21.14 | 13.82 | 8.94 |
| Claude | 23.58 | 13.01 | 5.69 |

**Panel H. Meals containing >50 g of carbohydrates - positive relative insulin-dose difference (%)**

| **Model** | **Meals with positive relative insulin-dose difference >10%, %** | **Meals with positive relative insulin-dose difference >20%, %** | **Meals with positive relative insulin-dose difference >30%, %** |
| --- | --- | --- | --- |
| ChatGPT | 4.17 | 0.00 | 0.00 |
| Gemini | 0.00 | 0.00 | 0.00 |
| DeepSeek | 4.17 | 0.00 | 0.00 |
| Claude | 12.50 | 8.33 | 4.17 |

Note. mean absolute theoretical insulin-dose differences were calculated by dividing the mean absolute carbohydrate-counting error by the insulin-to-carbohydrate ratio (ICR). Relative insulin-dose difference was calculated as (AI-estimated carbohydrate content - clinicians-derived reference carbohydrate content) / clinicians-derived reference carbohydrate content × 100. Because one meal in the overall dataset had a clinicians-derived reference carbohydrate value of 0 g, percentage-based relative insulin-dose calculations (Panels E-G) were performed on 123 meals overall and on 24 meals in the >50 g subgroup. “Theoretical excess insulin” refers to carbohydrate overestimation only, that is, cases in which the AI-estimated carbohydrate content exceeded the clinicians-derived reference value. Percentage thresholds (>10%, >20%, and >30%) were calculated using all meals with clinicians-derived reference values >0 as denominator. Meals containing >50 g of carbohydrates were analyzed separately because this was the subgroup in which between-model divergence was most evident.
