# Supplementary material for: Accuracy of ChatGPT, Gemini, Claude and DeepSeek in Carbohydrate Counting
Source: Diabetes Obes Metab. 2026 Apr 13;28(7):5627–36. doi: 10.1111/dom.70747 (PMC13243987; doi:10.1111/dom.70747)
Supplement: Supplementary file 8 — Table S5: Errors ChatGPT table. [file DOM-28-5627-s005.docx]

**Supplementary table 5.** Errors ChatGPT table.

- **Panel A: Errors frequency**

| Category | Count | % of ChatGPT errors | % of 124 meals |
| --- | --- | --- | --- |
| Reference discrepancy (expert) | 10 | 33.3 | 8.1 |
| Generic/average values (no specific match) | 6 | 20.0 | 4.8 |
| Over/underestimation (magnitude) | 5 | 16.7 | 4.0 |
| Other/unspecified | 5 | 16.7 | 4.0 |
| Brand-specific data not used | 2 | 6.7 | 1.6 |
| Instruction/data constraints not respected | 1 | 3.3 | 0.8 |
| Raw vs cooked mismatch | 1 | 3.3 | 0.8 |

- **Panel B: Errors.**

| N | Meal description | Error text | Category |
| --- | --- | --- | --- |
| 1 | 200 ml of semi-skimmed milk (Parmalat) + 3 rusks (7 g each) with 10 g apricot jam per slice | Error: generic estimate | Generic/average values (no specific match) |
| 5 | 50 g packaged fruit tart (Mulino Bianco) | Error: He did not follow the specific instructions given to him correctly | Instruction/data constraints not respected |
| 8 | 1 apple (120 g) + 1 packet wholemeal crackers (25 g Misura) | Error: ChatGPT relied only on the commercial label, without adapting it to the clinical criteria used by experts who subtract part of the fibers and consider only the truly digestible carbohydrates. | Brand-specific data not used |
| 13 | 1 slice wholemeal bread (40 g) with 15 g strawberry jam + 1 apple (100 g) | Error: Used unspecified average values ​​for bread and jam | Generic/average values (no specific match) |
| 14 | 150 g whole plain yogurt + 30 g whole muesli | Error: used unspecified average values ​​and reports lack of exact data from official label or databases | Generic/average values (no specific match) |
| 16 | 200 ml instant barley coffee (prepared with water and 1 teaspoon sugar) + 2 rusks (14 g total | Error: Overestimate the carbohydrate content of instant barley, using 3g/100ml instead of 1.5g/100ml | Over/underestimation (magnitude) |
| 17 | 200 ml soy milk (Alpro) + 20 g whole cereals | Error: generic estimate, without taking the brand into account | Brand-specific data not used |
| 18 | 200 ml tropical juice + 1 slice bread (50 g) with 10 g jam | Error: generic estimate | Generic/average values (no specific match) |
| 21 | 1 banana (140 g) + 3 classic rusks (30 g) | Error: You used higher average values ​​for bananas and toast. Banana sugar content varies depending on variety and ripeness. | Generic/average values (no specific match) |
| 23 | 200 ml orange juice + 1 slice wholemeal bread (50 g) with 30 g cream cheese (Certosa) | Error: generic estimate, simple rounding, differences in the details of the products considered | Generic/average values (no specific match) |
| 38 | 100 g potato gnocchi with buffalo mozzarella (70 g) and tomato sauce (80 g) + 5 g parmesan | The mistake is to use conservative values; the expert probably considered packaged dumplings rich in starch and sauce with additional ingredients. | Reference discrepancy (expert) |
| 43 | 250 ml vegetable broth with 30 g semolina pasta + 1 banana (100 g) | The error may be due to several factors:  1. Different nutritional databases: Sources may vary slightly in the carbohydrate values ​​indicated for each food.  2. Calculation method: I considered raw pasta, while the expert might consider cooked pasta, which has fewer carbohydrates per weight (because it absorbs water).  3. Type and exact weight of banana: I used a standard average value, but different banana varieties and degree of ripeness affect the sugar content.  4. Broth: I entered an approximate value, but it could be overlooked or calculated differently.  5. Possible rounding: The expert may have used more precise values ​​and different roundings. | Reference discrepancy (expert) |
| 60 | 100 g grilled beef + 150 g Brussels sprouts | Summary of my error: I estimated slightly higher (using a wider range), but the expert's assessment is correct for sprouts cooked plain, without additives. | Reference discrepancy (expert) |
| 70 | 80 g cooked rice salad with tuna and olives + 80 g banana | Error: The carbohydrate count of the "cooked" product is questionable. | Raw vs cooked mismatch |
| 72 | 80g di omelette con 150 g carrots e 40 g of white bread | Error: Evaluating the average nutritional values ​​of croutons without considering the specific types of croutons and the type of pear | Other/unspecified |
| 81 | 150 g white pizza | Error: Underestimating the carbohydrate density of white pizza, possibly confusing it with lighter bread or focaccia. | Over/underestimation (magnitude) |
| 83 | 150 g beef meatballs with 100 g mashed potatoes | Error: underestimating the ingredients of the meatballs and underestimating the puree, which has a higher glycemic density due to the processing | Over/underestimation (magnitude) |
| 92 | 80 g couscous with 20 g peppers, 20 g zucchini, 20 g onions | Error: Underestimating the carbohydrates in raw couscous by using a value that is too low compared to its actual content. | Over/underestimation (magnitude) |
| 93 | 100 g chicken curry with 50 g spinach | Error: considering the raw weight instead of the cooked weight | Other/unspecified |
| 94 | 150 g banana | Error: Bananas can vary greatly in sugar content depending on: variety, ripeness, and the specific data source used by the expert. | Reference discrepancy (expert) |
| 94 | 150 g banana | Error: Bananas can vary greatly in sugar content depending on: variety, ripeness, and the specific data source used by the expert. | Reference discrepancy (expert) |
| 95 | 200 ml pear juice (Zueg) | Error: A generic average value was used and not the exact value of the specific product | Other/unspecified |
| 99 | 20 g cereal bar (chocolate, Special K) | Error: A generic media from international nutritional databases was used. | Other/unspecified |
| 100 | 40 g white bread with 10 g quince jam | Error: It was used average values ​​that were a little lower than the specific ones of the product used by the expert | Reference discrepancy (expert) |
| 103 | 100 g banana and 15 g biscuits (Oro Saiwa) | Error: Bananas can vary greatly in sugar content depending on: variety, ripeness, and the specific data source used by the expert. | Reference discrepancy (expert) |
| 105 | 200 ml pear juice (Santal) and 14 g rusks (Mulino Bianco) | Error: Underestimating juice content by using a generic value instead of the product-specific value | Over/underestimation (magnitude) |
| 108 | 50 g tart | Error: he rated a homemade tart with less sugar while the expert rated an industrial tart | Reference discrepancy (expert) |
| 114 | 150 g banana + 15 g peanut butter | Error: Bananas can vary greatly in sugar content depending on: variety, ripeness, and the specific data source used by the expert. | Reference discrepancy (expert) |
| 115 | 150 ml semi-skimmed milk + 30 g wholemeal biscuits (Misura) | Error in calculating wholemeal biscuits, chat gpt 68 g cho because it uses fat secret (website) while the label says 59 | Other/unspecified |
| 122 | Fruit smoothie (150 ml milk + 100 g banana + 10 g honey) | Error: Bananas can vary greatly in sugar content depending on: variety, ripeness, and the specific data source used by the expert. | Reference discrepancy (expert) |
